# Using Community-Level Prevalence of *Loa loa* Infection to Predict the Proportion of Highly-Infected Individuals: Statistical Modelling to Support Lymphatic Filariasis and Onchocerciasis Elimination Programs

**DOI:** 10.1371/journal.pntd.0005157

**Published:** 2016-12-01

**Authors:** Daniela K Schlüter, Martial L Ndeffo-Mbah, Innocent Takougang, Tony Ukety, Samuel Wanji, Alison P Galvani, Peter J Diggle

**Affiliations:** 1 CHICAS, Lancaster Medical School, Lancaster University, Lancaster, United Kingdom; 2 Department of Epidemiology of Microbial Diseases, Yale School of Public Health, New Haven, Connecticut, United States of America; 3 Foundation for Health Research & Development, Department of Public Health, University of Yaoundé 1, Yaoundé, Cameroon; 4 World Health Organisation, Geneva, Switzerland; 5 Parasites and Vectors Research Unit, Department of Microbiology and Parasitology, University of Buea, Cameroon; 6 Research Foundation in Tropical Medicine and Environment, Buea, Cameroon; Imperial College London, Faculty of Medicine, School of Public Health, UNITED KINGDOM

## Abstract

Lymphatic Filariasis and Onchocerciasis (river blindness) constitute pressing public health issues in tropical regions. Global elimination programs, involving mass drug administration (MDA), have been launched by the World Health Organisation. Although the drugs used are generally well tolerated, individuals who are highly co-infected with *Loa loa* are at risk of experiencing serious adverse events. Highly infected individuals are more likely to be found in communities with high prevalence. An understanding of the relationship between individual infection and population-level prevalence can therefore inform decisions on whether MDA can be safely administered in an endemic community. Based on *Loa loa* infection intensity data from individuals in Cameroon, the Republic of the Congo and the Democratic Republic of the Congo we develop a statistical model for the distribution of infection levels in communities. We then use this model to make predictive inferences regarding the proportion of individuals whose parasite count exceeds policy-relevant levels. In particular we show how to exploit the positive correlation between community-level prevalence and intensity of infection in order to predict the proportion of highly infected individuals in a community given only prevalence data from the community in question. The resulting prediction intervals are not substantially wider, and in some cases narrower, than the corresponding binomial confidence intervals obtained from data that include measurements of individual infection levels. Therefore the model developed here facilitates the estimation of the proportion of individuals highly infected with *Loa loa* using only estimated community level prevalence. It can be used to assess the risk of rolling out MDA in a specific community, or to guide policy decisions.

## Author Summary

Lymphatic Filariasis (LF) is caused by parasitic worms which live in the lymphatic system. Though several body parts may be affected, LF characteristically leads to the enlargement of limbs, causing pain, physical disability and social stigma. Onchocerciasis (river blindness) is caused by similar worms which inhabit the subcutaneous tissue and lead to disturbing skin lesions, visual impairment and blindness. Onchocerciasis and LF affect over 146 million people, and have been targeted for elimination as public health problems by the World Health Organisation, using mass drug administration (MDA). Although the drugs used in MDA are generally well tolerated, individuals who are highly co-infected with *Loa loa* are at risk of experiencing serious adverse events. In order to inform decisions on whether MDA can be safely administered in a community, we investigate the relationship between community level prevalence of *Loa loa* and individual infection levels through the development of a statistical model. We find a strong positive correlation and show how this can be exploited to estimate the proportion of individuals highly infected with *Loa loa* without the need for data on individual infection levels. The model therefore provides a method to assess the risk of rolling out MDA in a specific community, or to guide policy decisions.

## Introduction

Lymphatic Filariasis (LF) and Onchocerciasis are parasitic helminth diseases that constitute a serious public health issue in tropical regions [1]. LF causes one of the highest global burdens of all vector-borne diseases, with an estimated 120 million people infected in 83 countries [1]. As LF, onchocerciasis is a major human disease which affects an estimated 26 million people in 34 countries [1, 2]. Both diseases have been targeted for elimination by the World Health Organisation (WHO), using mass drug administration (MDA) [1, 3]. The LF elimination program is based on annual mass administration of a single dose of diethylcarbamazine or ivermectin combined with albendazole [1, 4], while the onchocerciasis elimination program is based on mass administration of ivermectin only [1]. Although both medications are generally considered to be safe, individuals who are heavily co-infected with *Loa loa* parasites are at risk of developing severe, even fatal, adverse reactions to either drug [5–8]. Heavily co-infected individuals are more likely to be found in communities with high prevalence [5]. This observation has led to a requirement that precautionary measures be implemented before roll-out of LF and onchocerciasis MDA to communities in *Loa loa* high-prevalence areas [9, 10].

In this study we investigate the distribution of *Loa loa* microfilarial loads in communities. To address this problem we first develop a model for the variation in parasite count between individuals within a community, and explore how the parameters of this distribution co-vary with each other and with community-level covariates.This study builds on previous work which also looked at the distribution of *Loa loa* microfilarial loads in communities [11]. In contrast to this earlier work, we allow the relationship between community-level prevalence and mean intensity of infection to vary stochastically between communities. By exploiting the correlation between these two community-level features we are able to predict the proportion of a community whose parasite count exceeds a policy-determined threshold when only prevalence data are available for the community in question. We can also quantify the uncertainty associated with this prediction. This can help inform decisions on whether LF or onchocerciasis MDA can be safely administered in a community, given only an estimate of *Loa loa* prevalence. Thus it can be used to guide policy decisions regarding the targeting of communities for LF and onchocerciasis MDA, especially for hypo-endemic communities where treatment strategies still need to be defined [12].

## Materials and Methods

### Data Sources

#### Epidemiological data

**Fig 1:**
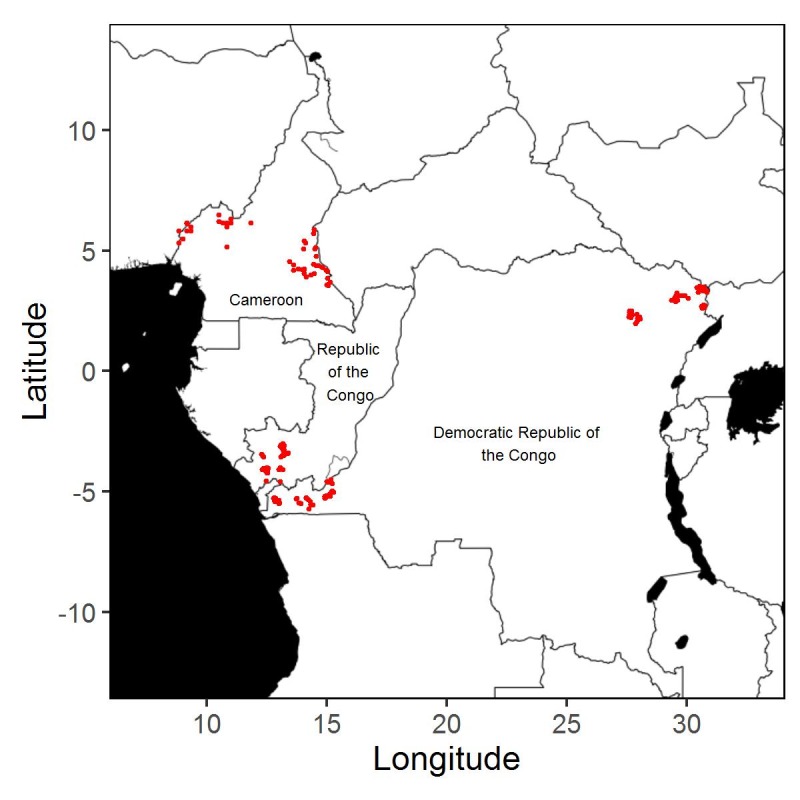
Map showing the locations of the 222 villages for which data are available.

**Fig 2:**
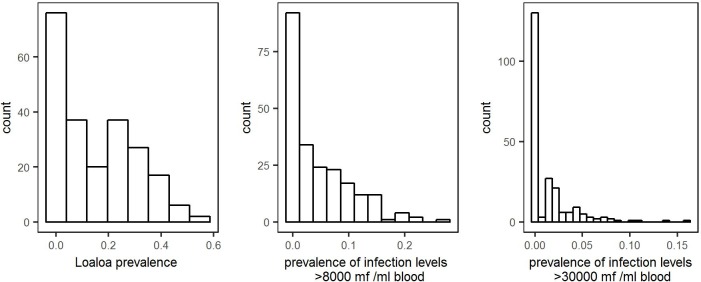
Distributions of *Loa loa* prevalence (left panel), the proportion of individuals with infection levels greater than 8,000 microfilariae per ml blood (centre panel) and the proportion with infection levels greater than 30,000 microfilariae per ml blood (right panel), in the data from all 222 villages.

The epidemiological data available were obtained from two field studies in village communities (henceforth villages) conducted in the West and East provinces of Cameroon between July and August 2001 [13] as well as in the Republic of the Congo and the Bas-Congo and Orientale provinces of the Democratic Republic of the Congo (DRC) between January and May 2004 [14]. Fig 1 shows the locations of all sampled villages. The Cameroon data contain the estimated *Loa loa* infection level (parasites/ml of blood) in samples from between 24 and 229 individuals in a total of 73 villages [13]. The data from the Republic of the Congo and the DRC contain the same information from between 27 and 102 individuals in a total of 149 villages, together with the bio-ecological zones of each village (forest, mosaic forest savannah, savannah) [14]. Collectively, the two data-sets cover 19 049 individuals from 222 villages. The distributions, across all 222 villages, of *Loa loa* prevalence, the proportion of individuals with infection levels greater than 8,000 microfilariae per ml blood and the proportion with infection levels greater than 30,000 microfilariae per ml blood are given in Fig 2.

#### Environmental data

We obtained the following environmental data to be considered as candidate covariates:

- Average annual rainfall and temperature at 1km resolution from the WorldClim database (http://www.worldclim.org) for the years 1950–2000 and 1950–2001, respectively;
- Elevation at 90m resolution from the Shuttle Radar Topography Mission;
- Maximum normalised-difference vegetation index (NDVI) for 2004 and standard deviation of NDVI for 2005 at 250m resolution acquired from the Moderate-Resolution Imaging Spectro-radiometer at the village coordinates and as an average over a 5km radius around the village coordinates;
- Percentage of forest cover at 300m resolution acquired from GlobCover for the years 2004–2006, averaged over a 5km radius around the village coordinates.

### Statistical Model

Let *Y* denote the parasite count/ml blood for a randomly selected person in a village, and *F*(*y*) the probability that *Y* ≤ *y*. Then, the village-level prevalence is *ρ* = 1 − *F*(0). Our model for *F*(*y*) incorporates a discrete probability mass 1 − *ρ* at *y* = 0, and continuously varying values conditional on *y* > 0. Hence,

$$F(y;\rho ,\lambda ,\kappa )=\left\{ \begin{array}{l} 1-\rho \;y=0\\ (1-\rho )+\rho G(y;\lambda ,\kappa )\;y>0 \end{array}\right.,$$ (1)

where *y* is the parasite load/ml blood, *ρ* the village-level prevalence and *G*(*y*; *λ*, *κ*) a continuous parametric distribution with parameters *λ* and *κ*.

**Fig 3:**
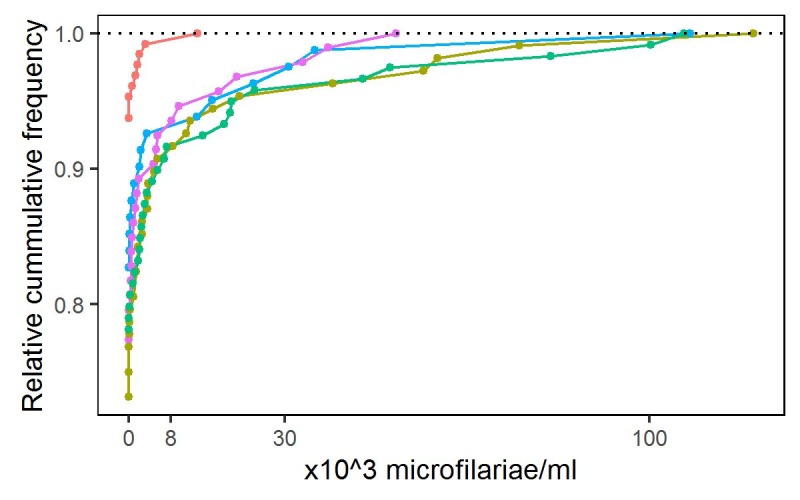
Empirical cumulative distributions of the parasite levels in individuals from five randomly selected villages.

**Fig 4:**
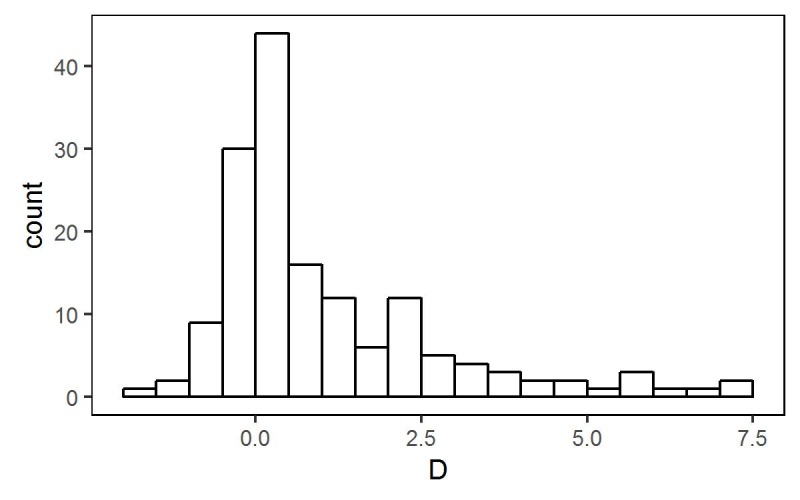
Log-likelihood-ratio statistic *D* = 2(*L*_*w*_ − *L*_*g*_), for 156 villages where *L*_*w*_ and *L*_*g*_ denote maximised log-likelihoods for Weibull and Gamma fits, respectively.

The empirical distributions of parasite load in individual villages (Results, Fig 3) suggest as suitable candidates for *G*(*y*; *λ*, *κ*) either the Gamma or Weibull family, with the Weibull generally giving the better fit (Results, Fig 4) and, in either case, κ<1. This is consistent with the general observation that parasite load distributions in parasitic infected communities are over-dispersed with a small proportion of individuals harbouring a large proportion of the parasite load within the population [15].

In fitting the model to the data from all 222 villages, we reduced the total number of parameters by assuming a constant value of *κ* (S1 Supplementary Material, section S.1). The resulting model for village *i* is

$$F(y;\rho_{i},\lambda_{i},\kappa )=\left\{ \begin{array}{l} 1-\rho_{i}\;y=0\\ (1-\rho_{i})+\rho_{i}[1-\exp\left(-{\left(\frac{y}{\lambda_{i}}\right)}^{\kappa }\right)]\;y>0 \end{array}\right..$$ (2)

In (2) we then modelled the variation amongst the *ρ*_*i*_ and the *λ*_*i*_ by logistic and log-linear mixed effects regressions, respectively. Hence,

$$\log\left(\frac{\rho_{i}}{1-\rho_{i}}\right)={d\prime }_{i}\alpha +U_{i}\;:\;i=1,\ldots \;n,$$ (3)

and

$$\log\left(\lambda_{i}\right)=d\prime_{i}\beta +V_{i,}\;:\;i=1,\ldots \;n,$$ (4)

where the *d*′_*i*_ are vectors of covariates and the (*U*_*i*_,*V*_*i*_) are random effects, bivariate Normally distributed with means zero, variances *σ*_*U*_^2^ and *σ*_*V*_^2^, and correlation *ϕ*. The values of *U*_*i*_ and *V*_*i*_ are realised independently in different villages.

### Inference

We estimate the model parameters by the method of maximum likelihood. For data from samples of size *n*_*i*_ in each of *m* villages *i*, let *y*_*ij*_ denote the parasite count/ml for individual *j* in village *i* and *z*_*i*_ the number of *y*_*ij*_ > 0. Hence,

$$L(U_{i},V_{i})=(n_{i}-z_{i})\;\log\left(1-\rho_{i}\right)+z_{i}\;\log\;\rho_{i}+\sum_{j=1}^{n_{i}}\log\;g(y_{ij};\lambda_{i},\kappa ),$$ (5)

where *ρ*_*i*_ and *λ*_*i*_ are defined by (3)and (4), respectively. The log-likelihood for the observed data is then

$$L(\alpha ,\beta ,\kappa ,{\sigma_{U}}^{2},{\sigma_{V}}^{2},\phi )=\sum_{i=1}^{m}\log\left\{\int \int \;\exp\{L_{i}(u,v)\}\;h(u,v;{\sigma_{U}}^{2},{\sigma_{V}}^{2},\phi )du\;dv\right\},$$ (6)

where *h*(∙) denotes the bivariate Normal density of (*U*_*i*_,*V*_*i*_). We evaluate the integrals on the right-hand side of(6) numerically, using a quasi-Monte Carlo method. Details are given in S1 Supplementary Material, section S.2.

### Model Development

We first fitted separate models of the form (1) to data from each village, specifying either a Gamma or Weibull distribution for *G*(∙) and compared the maximised values of the log likelihood, which suggested that the Weibull gave the better fit (Results, Fig 4). We therefore fitted the single model, as defined by Eqs(2), (3), and(4), to the complete data-set, estimating the parameter vector (*α*, *β*, *κ*, *σ*_*U*_^2^, *σ*_*V*_^2^, *ϕ*) by maximising the log-likelihood defined at Eq(6). To identify potentially important covariate effects, we used a combination of informal graphical methods, subject-matter knowledge from the literature and significance testing based on the asymptotic chi-squared distribution of the maximised log-likelihood-ratio *D*, defined as twice the difference between maximised log-likelihoods for models with and without the covariate in question.

### Prediction

Our goal is to predict, for each village, the proportion of inhabitants whose level of infection exceeds a policy-specified threshold, *c*, given only the number, *Z*, of infected individuals in a random sample of size *n*. Hence, our target for prediction is *T* = *ρ*{1 − *G*(*c*)}. A relevant value for *c* is one that is considered to pose a material risk of experiencing an adverse reaction to prophylactic administration of anti-filarial medication. Current debate is around whether 8000 or 30 000 microfilariae/ml is the more appropriate. We cannot calculate *T* directly, because we do not observe the random effects *U* and *V*, for the village in question. For prediction of *T*, we therefore calculate the plug-in predictive distribution of *T* i.e. the distribution of *T* given *n*, *Z*, model parameter estimates and, if relevant, covariate values. This distribution is analytically intractable. However, given values for the model parameters and for the covariates from a particular village, *ρ* and *G*(*c*) are explicit functions of *U* and *V*, respectively, i.e. *T* = *T*(*U*,*V*). We therefore simulate samples from the conditional distribution of (*U*,*V*) given the data. We then transform each such sample according to the formula for *T*(*U*,*V*) to obtain samples from the required predictive distribution. By repeating this process a sufficiently large number of times, we obtain arbitrarily close approximations to the required predictive distributions. The algorithm that we used for this is described in S1 Supplementary Material, section S.3.

In order to investigate whether the uncertainty in estimating the model parameters materially affects the predictive distribution of *T*, we carry out a sensitivity analysis by using samples drawn from the sampling distribution of these parameters rather than their point estimates. For this sensitivity analysis we assume that the parameters follow a multivariate Normal distribution whose mean is given by the set of point estimates and whose variance-covariance matrix is the inverse of the observed Fisher information matrix. For each such sample of parameter values, we then simulate a draw from the corresponding predictive distribution of (*U*, *V*), and hence from the predictive distribution of *T*.

Predictions to guide policy can also be made. A potential policy-relevant question is: what can we say about the proportion of highly infected people in hypothetical communities with true prevalence, *p*? For a given threshold *c* and estimated values of the model parameters, the proportion of highly infected people, *T*, is a function of two random variables, *U* and *V*, i.e. *T* = *T*(*U*,*V*). Knowing *p* is equivalent to knowing the value of *U*, say *U* = *u*. Hence, the relevant predictive distribution to answer the stated question is the conditional probability distribution of *T*(*U*,*V*) given *U* = *u*. To simulate samples from *T(U*,*V)* given *U = u*, we simulate samples from the distribution of *V* given *U = u* and then transform each sample according to the formula for *T(U*,*V)*. This is distinct from the prediction of the proportion of highly infected individuals in a specific community based on the observed prevalence in a sample, for which samples from the conditional distribution (*U*,*V | Z*, *n*) have to be simulated before calculating *T*(*U*,*V*) given *Z* and *n* to take into account the uncertainty around the observed prevalence.

## Results

For 67 of the villages, the data contain fewer than 4 individuals found to be infected. We excluded these from the first-stage analysis reported in the section immediately below, but included them thereafter.

### Single-Village Analysis

We first considered which of the Gamma and Weibull distributions gave the better fit to the data by comparing the maximised values of their log-likelihoods. Fig 4 shows the distribution of *D* = 2(*L*_*w*_ − *L*_*g*_) over the 156 villages with 4 or more positive infection levels, where *L*_*g*_ and *L*_*w*_ are the maximised values of the log-likelihoods assuming Gamma and Weibull distributions, respectively. The preponderance of positive values suggests that the Weibull gives the better fit.

**Fig 5:**
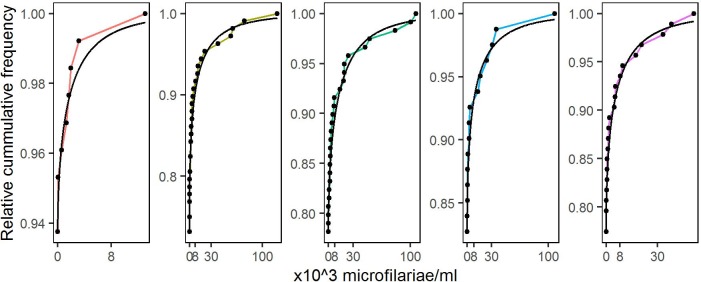
Empirical (coloured lines and dots) and fitted (black lines) cumulative distributions of parasite levels for the five randomly selected villages shown in Fig 3.

Fig 5 compares the empirical and fitted cumulative distribution functions for the same five randomly selected villages as in Fig 3. The convex shape indicates that in each case *κ*_*i*_ < 1. Empirical distributions of the maximum likelihood estimates for parameters of the Weibull model, estimated separately for each of the 156 villages, are given in Figure Ain the S1 Supplementary Material.

### Linking the Villages

To link the villages and reduce the number of model parameters accordingly, we modelled the *ρ*_*i*_ and *λ*_*i*_ using the mixed-effects regression framework set out in Eqs (2), (3) and (4). We first fitted each of the candidate covariates separately, then developed multiple regression models for *ρ*_*i*_ and *λ*_*i*_ by forward selection. Following [16], we fitted MaxNDVI and elevation as split-line regressions. In the case of MaxNDVI, the rationale is that NDVI, considered as a proxy for green-ness of vegetation, is saturated beyond a value of 0.8. For elevation, the rationale is that the disease vector cannot breed successfully above 1000 metres elevation. Following [17], we used maximum NDVI and standard deviation of NDVI averaged over a 5km radius around the village as candidate covariates rather than the measurements at the village coordinates themselves, in order to account for the flight range of the vector.

**Fig 6:**
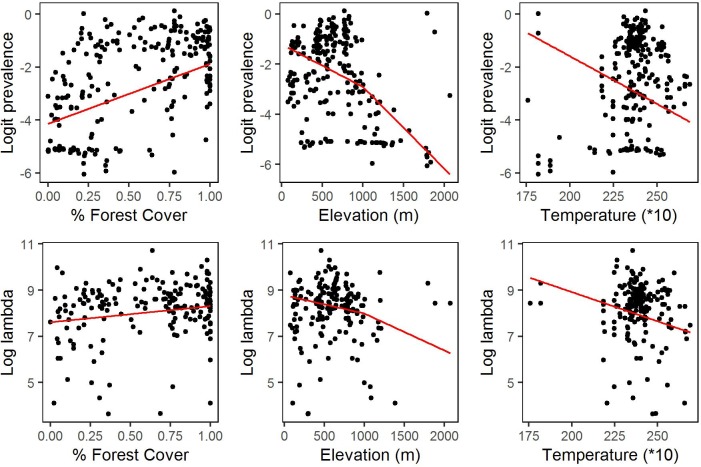
Village-specific estimates of log(ρ/(1-ρ)) (upper row) and of logλ (lower row) plotted against percent forest cover within a 5km radius (left column), elevation (centre column) and average temperature (right column). Red lines show the fitted contributions to the multivariate regression model defined by **E**qs (2), (3) and (4).

Percentage forest cover gave the smallest *p*-value for the likelihood ratio test against a model with no covariates (Table 1). Adding elevation gave a likelihood ratio statistic *D* = 19.91 on 4 degrees of freedom, corresponding to a *p*-value of 0.0005. Adding temperature to the model that included both forest cover and elevation gave a likelihood-ratio statistic *D* = 9.7 on 2 degrees of freedom, corresponding to a *p*-value of 0.0079. Adding further covariates gave minimal, non-significant improvements in the likelihood. Therefore, the covariates in our provisional model are percentage forest cover, elevation and temperature. Although these covariates show statistically significant associations with prevalence and intensity of infection, they explain only small proportions of the parameter variation between villages (Fig 6).

**Table 1:** Log-likelihood-ratio statistics, D, degrees of freedom, df, and p-values, p, for candidate covariates in univariable analysis.

Variable	*D*	*df*	*p*
Max(NDVI)	11.93	2	0.0026
Sd(NDVI)	12.16	2	0.0023
Elevation	35.72	4	3.3*10^−7^
Rainfall	4.41	2	0.35
% Forest Cover	68.25	2	1.55*10^−15^
Temperature	13.89	2	0.00096

Table Ain S1 Supplementary Material, section S.2 gives parameter estimates, standard errors and 95% confidence intervals for the provisional model, and for a simpler model without covariates. In either case, there is a substantial positive correlation between the village-level random effects *U*_*i*_ and *V*_*i*_. This is fundamental to the ability of village-level empirical prevalence to predict the proportion of highly-infected people in a specific village.

### Prediction

**Fig 7:**
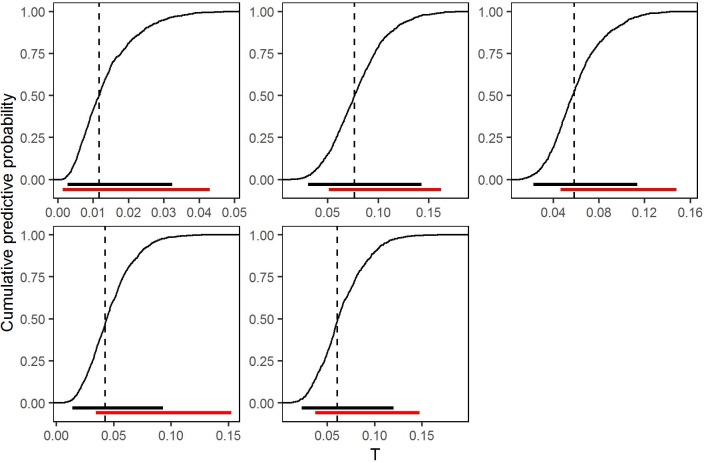
Cumulative predictive distributions of *T*, the proportion of village residents with parasite count greater than *c* = 8000/ml, for the five randomly selected villages shown in Figs 3 and 5, based on the fitted model without covariates. Dashed vertical lines show the point predictions. Black horizontal lines show equal-tailed 95% plug-in predictive intervals. Red horizontal lines show the corresponding 95% confidence intervals for the true proportions based on the binomial sampling distribution of the observed numbers of individuals in each village with parasite count greater than *c* = 8000/ml.

Using the simpler model without covariates, we calculated the cumulative predictive distributions of *T* = *ρ*{1 − *G*(8000)} for the same five villages featured in Figs 3 and 5 (Fig 7). Models with and without covariates gave very similar predictive distributions, consistent with the earlier observation that the covariates, although statistically significant, explain minimal empirical variation (S1 Supplementary Material, section S.4). Thus, in practice, there is little or no advantage in using the currently available covariates to predict *T*.

We use the median of the predictive distribution as a point prediction for *T*, and the interval between the 2.5% and 97.5% quantiles of the predictive distribution as a 95% predictive interval (Fig 7). For comparison, Fig 7 also shows 95% confidence intervals for *T* derived from the observed proportions of sampled individuals whose level of infection exceeded *c* = 8000 parasites per ml of blood. Determining individual levels of infection is precisely what we wish to avoid, because of the associated practical difficulties of doing so routinely in the field. It is therefore encouraging that model-based predictive intervals using only the empirical prevalence overlap with the corresponding binomial confidence intervals using measured infection levels, but are not substantially wider, and in some cases are actually narrower. Table Bin S1 Supplementary Material, section S.3 gives point predictions of *T* and associated 95% predictive intervals for each of the 222 villages in our data.

**Fig 8:**
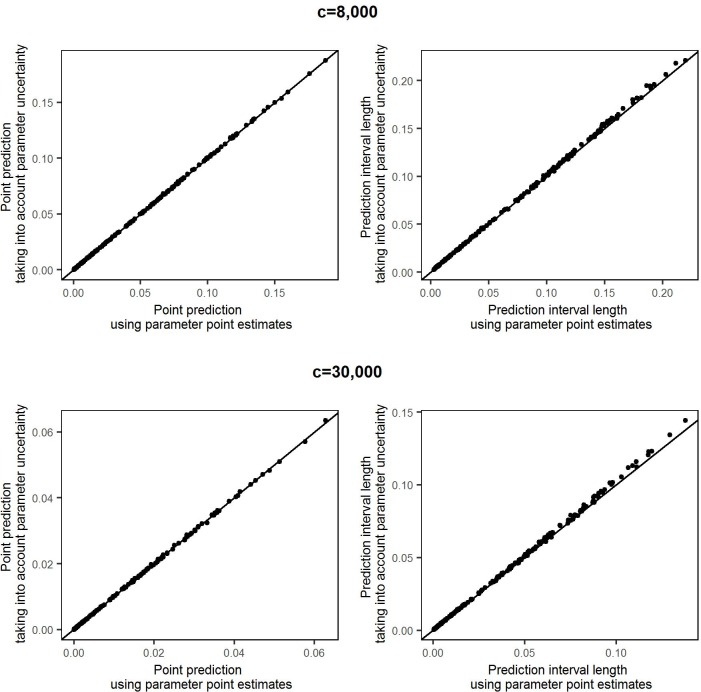
Comparison of predictions using the reported point estimates of model parameters and taking into account the uncertainty around the estimated parameter values. The upper row shows point predictions (left column) and prediction interval lengths (right column) for a high infection threshold *c* = 8,000. The lower row shows the corresponding comparsions for a threshold *c* = 30,000.

The predictive accuracy illustrated in Fig 7 relies on the fit of the probability model. To verify the insensitivity of our results to uncertainty in estimation of the model parameters, we repeated the predictions for all 222 villages using samples from the multivariate normal distribution of the parameter estimates rather than their point estimates (see Methods section Prediction). We drew 10,000 samples, which is the same number that we generated from the plug in predictive distribution when using point estimates of the parameters. Fig 8 shows the point predictions and the lengths of the prediction intervals, comparing the results when using point estimates and when accounting for the uncertainty in the parameter values. The differences in the point predictions obtained by the two approaches are negligible, and the lengths of the prediction intervals only increase marginally when the corresponding plug-in intervals are already wide, i.e. in villages with very small samples. These results are also given in Table B in the S1 Supplementary Material, section S.3.

**Fig 9:**
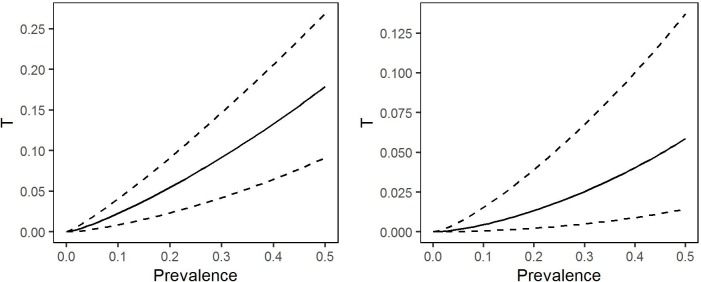
Selected quantiles of the conditional probability distribution of the proportion of individuals with intensity of infection greater than *c* = 8,000 parasites per ml of blood (left-hand panel) or *c* = 30,000 parasites per ml of blood (right-hand panel), as functions of village-level prevalence. The median (solid line) provides a “best guess” of the relationship between prevalence and proportion of highly infected individuals; the 2.5% and 97.5% quantiles indicate the imprecision of this best guess.

Again using the covariate free version of the model, we also calculated the conditional distribution of *T*(*U*,*V*) given *U* = *u* to answer the question: what can we say about the proportion of highly infected people in a hypothetical village as a function of its true prevalence. The left-hand panel of Fig 9 shows selected quantiles of this distribution over a range of values of prevalence, based on the fitted model without covariates. The median curve shows, for example, that a 20% prevalence corresponds to an estimated proportion 0.055 of individuals with parasite counts greater than 8,000 per ml of blood, and that the associated 95% prediction interval runs from 0.023 to 0.091. The right-hand panel of Fig 9 shows that using the higher threshold value *c* = 30 000, a 20% prevalence corresponds to an estimated proportion 0.0134 of highly infected individuals, with associated 95% prediction interval running from 0.002 to 0.039.

## Discussion

Modelling the relationship between village-level prevalence and the distribution of infection intensity enables prediction of the proportion of heavily infected individuals in a sampled community, using only the observed prevalence in a sample of individuals. Furthermore, these intervals are almost as precise as would be predictions based on directly ascertaining the proportion of highly infected individuals.

This work builds on previous investigations into the distribution of *Loa loa* infection intensities in communities [11]. In this earlier work a zero-truncated negative binomial distribution is fitted to data on microfilarial loads in individual communities. Although technically different, the earlier model is similar in spirit to ours, except that it models the relationship between prevalence and mean intensity of infection deterministically. Consequently, it cannot be used calculate the uncertainty associated with village-level predictions, but only to predict the “on average” relationship between the proportion of highly infected individuals in a village and the true prevalence in the village.

As we have shown in Fig 9, our model can also be used for this purpose, and the relationships that we find (Fig 9) are very similar to those shown, albeit without any indications of their uncertainty, in Fig 4 of [11].

If the village-specific random effects *U*_*i*_ and/or *V*_*i*_ exhibited smooth spatial variation, data from multiple villages could be combined to improve the precision of predictions for a given village. This would involve formulating and fitting a model for a bivariate spatial process *{U(x)*,*V(x)}*, where *x* denotes location, and the correlation between the elements of *U(x)* and/or *V(x)* at a pair of locations *x* and *x’* depends on the distance between *x* and *x’*. As a first, simpler step in investigating spatial effects, we fitted a version of the model treating study-site as a 5-level factor: 1) DRC Bas Congo, 2) DRC North-West, 3) the Republic of the Congo, 4) Cameroon West and 5) Cameroon East. The likelihood ratio statistic to compare this model to the model without covariates was *D* = 63.79 on 8 degrees of freedom (*p* < 0.001). However, as previously seen with the environmental covariates, although statistically significant the differences amongst the five study-sites explained little of the variation between villages (S1 Supplementary Material, section S.5). Parameter estimates, standard errors and 95% confidence intervals are included in Table Ain S1 Supplementary Material, section S.2.

The predictive methods developed here are intended to be practicable in field-work conditions. Our predictive algorithm runs on a lap-top computer using open-source software. In the simple version of the model without covariates, the field-worker would need only to record the number of positive and negative results from sampled individuals, choose a critical infection level *c* and either enter this information into their lap-top, or transmit it as a text-message for remote analysis. The algorithm would then output the predicted proportion of highly infected individuals as a point estimate, and the predictive distribution in graphical form. In the absence of covariates, it would also be feasible to develop a mobile phone implementation, using a table look-up in conjunction with pre-computed percentiles of the predictive distribution of *T* for each relevant combination of *c*, *n* and *z*.

If, in due course, either a spatial model or one with covariates were to be used, the computer running the predictive algorithm could be loaded with an accumulating set of geo-referenced prevalence data from previously sampled villages and any relevant covariate images covering the geographical region of interest.

A practical limitation to applying the model-based predictions in the field is the number of people of whom blood samples need to be taken in order to get predictions with satisfactory precision. The decisions regarding how large a proportion of highly infected individuals in a village is acceptable and the certainty with which this proportion is not exceeded, have to be made by the MDA program coordinators. This means that there can be no hard rules for the number of individuals that need to be tested in any one village. However, a feasible strategy is to first sample a manageable number of people, say 100, to be tested and calculate the relevant predictive distributions. Should the uncertainty around the resulting prediction be too large, additional individuals could be sampled and the prediction updated until a satisfactory precision in the predictions is reached. New developments in mobile microscopy, which facilitate the fast, automatic quantification of *Loa loa* microfilariae in whole blood using mobile phones, may lead to quicker and cheaper ways of testing more individuals and therefore obtaining higher precision in the predictions. Before routine use, it would be wise to recalibrate the model using data collected with mobile rather than classical microscopy. The first study using the mobile microscopy unit *CellScope* has shown strong correlation between *CellScope* counts and the counts generated by classical microscopy (94% specificity, 100% sensitivity) in 33 individuals in Cameroon [18]. Notwithstanding the small size of this first study, it would therefore seem unlikely that the specific form of microscopy would necessitate any fundamental change in the structure of the model. Note also that the predictions reported in the present paper are based solely on sample prevalence data, hence their validity for data collected by different technologies requires only that these technologies produce prevalence estimates that are in line with estimates obtained using classical microscopy. Nevertheless, our motivation for deriving a method of prediction that uses only prevalence information was that classical microscopy is relatively impractical for routine use in the field. Should newer technologies remove that practical constraint, the same model, with suitable re-calibration of the model parameters, can be used to make predictive inference that use all of the available data, with consequent gains in precision for fixed sample size.

Rigorous validation of our predictive inferences is not possible, as it would require data to be collected from villages with known true prevalence, which in turn would require every individual in each village to be tested. Informally, the qualitative agreement between our model-based 95% predictive intervals and binomial 95% confidence intervals based on the observed number and proportion of sampled individuals who are highly infected (Fig 7) is encouraging, because the binomial estimates, although potentially inefficient, are guaranteed to be unbiased. As a further partial validation, we have applied our model, without any re-estimation of its parameters, to further samples from 253 village communities in Cameroon, Gabon and Equatorial Guinea which were not used for the model development. To get a sense of the accuracy of these out-of-sample predictions, we predict the proportion of highly infected individuals and associated 95% predictive intervals for each of these villages and compare them with the observed proportion of highly infected individuals in the samples and the associated 95% confidence intervals based on the binomial sampling distribution. See S1 Supplementary Material, section S.6 (Figures E, F). The high degree of overlap between the model-based and binomial intervals illustrated in Fig 7 is generally maintained. The largest differences occur when high infection levels are observed in a small sample and the binomial confidence intervals are therefore relatively wide. In these cases, the model generally predicts the true proportion of highly infected individuals to be towards the lower end of the binomial confidence interval, and the model-based prediction interval is narrower than the binomial confidence interval. This illustrates a general phenomenon, that when data are sparse, parametric modelling assumptions can impart substantial gains in statistical efficiency by comparison with non-parametric methods.

In summary, using empirical data to evaluate the relationship between prevalence and intensity of infection, we developed a modelling tool that can be used for operational decision-making in the field to predict the proportion of a village community that is heavily infected with *Loa loa* using only empirical prevalence data for the community in question. The model can also be used to inform policy decisions regarding the safety of an MDA approach to treating lymphatic filariasis or onchocerciasis within specific sets of communities.

## Supporting Information

S1 Supplementary Material Additional figures, tables and technical details regarding the parameter estimation and target prediction. (PDF)

S1 Dataset Dataset containing infection level data. (CSV)

S2 Dataset Dataset containing covariate data. (CSV)
