# Supplementary material for: Using Community-Level Prevalence of Loa loa Infection to Predict the Proportion of Highly-Infected Individuals: Statistical Modelling to Support Lymphatic Filariasis and Onchocerciasis Elimination Programs
Source: PLoS Negl Trop Dis. 2016 Dec 1;10(12):e0005157. doi: 10.1371/journal.pntd.0005157 (PMC5132393; doi:10.1371/journal.pntd.0005157)
Supplement: S1 Supplementary Material — (PDF) [file pntd.0005157.s001.pdf]

Supplementary Material

accompanying manuscript:

"Using community-level prevalence of Loa loa infection to predict the proportion of highly-infected individuals: statistical modelling to support lymphatic filariasis elimination programs"

Daniela K Schlüter, Martial L Ndeffo-Mbah, Innocent Takougang, Tony Ukety, Samuel Wanji, Alison P Galvani, and Peter J Diggle

S.1 Data-analysis to support the adoption of a single Weibull shape parameter κ for all villages.

Figure A shows the empirical distributions of the maximum likelihood estimates, $\log\{\hat{\rho}_i/\{1-\rho_i)\}$, $\log(\hat{\lambda}_i)$ and $\hat{\kappa}_i$ estimated separately for each of the 156 villages that returned four or more positive results.

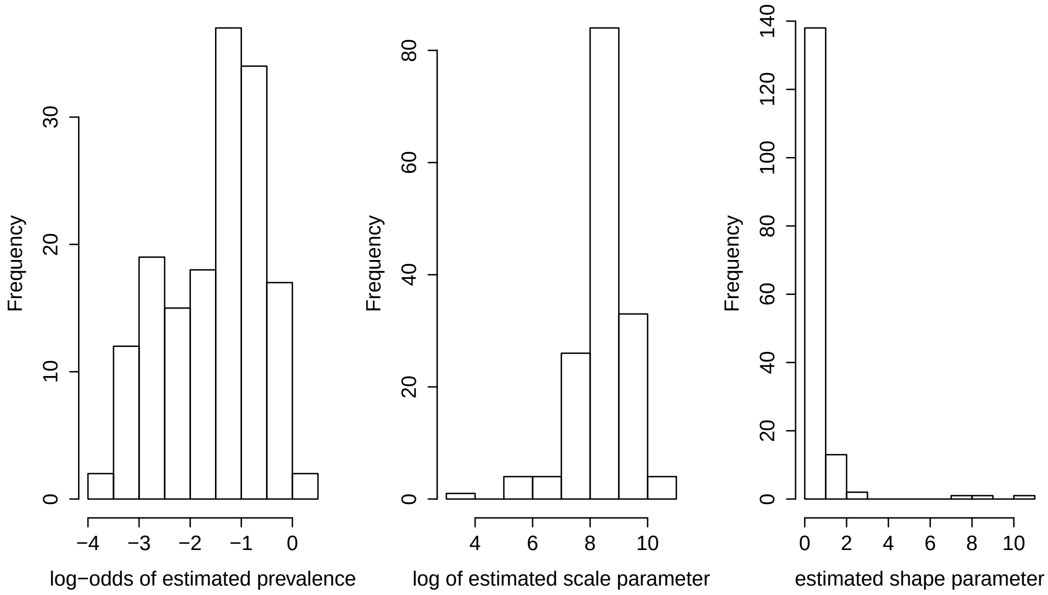

Figure A: Parameter estimates for the Weibull model fitted separately to each of 156 villages.

The empirical distributions of $\log\{\hat{\rho}_i/\{1-\rho_i)\}$ and of $\log(\hat{\lambda}_i)$ are unimodal and moderately negatively skewed, suggesting that linear mixed effects modelling on these transformed scales is not unreasonable. The empirical distribution of the $\hat{\kappa}_i$ is extremely skewed, with most values less than 1; also, the larger values of $\hat{\kappa}_i$ have larger associated standard errors. The likelihood-ratio statistic indicates a significant difference between a model with a single shape parameter κ, estimated as $\hat{\kappa} = 0.55$, and 156 separate parameters κ_i ($D = 239$ on 155 degrees of freedom, $p < 0.001$). However, estimates for the village-specific prevalences $\hat{\rho}_i$ and scale parameters $\hat{\lambda}_i$ were almost unchanged whether or not we estimated separate values of κ for each village (Figure B).

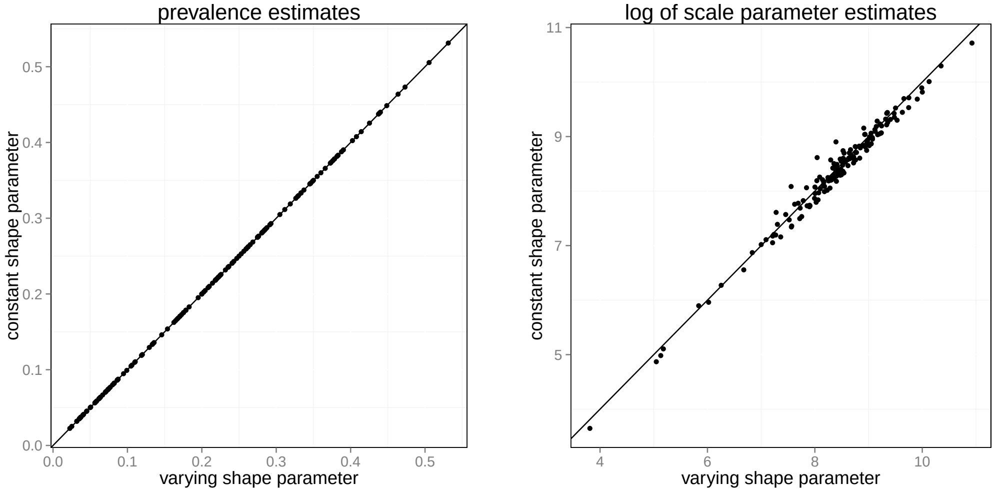

Figure B: Estimated village-specific prevalences ρ_i shape parameters λ_i for a common value of the shape parameter, $\hat{\kappa} = 0.55$, against the corresponding estimated values allowing village-specific estimates $\hat{\kappa}_i$.

S.2 Parameter estimation

Parameters were estimated using maximum likelihood in conjunction with a quasi-Monte Carlo algorithm to evaluate the integrals on the right-hand side of equation (6) (main article). The algorithm was implemented using a two-dimensional Halton sequence for the generation of the integration points. The number of points used was 1000. All the candidate covariates included in the model were centred. Maximum NDVI was centered at 0.8, elevation at 1000 and the remaining covariates at their observed means. Numerical maximisation of the likelihood was performed with the `R` function `nlminb()` which uses PORT routines for optimisation. These routines are based on a reverse-communication trust-region quasi-Newton method.

For the numerical optimisation, transformed parameters with unconstrained ranges were used. The correspondence between the transformed parameters θ, as listed in Table A, and the original parameterisation is as follows for the model without covariates: θ_1 is the intercept for the regression on the log-odds of prevalence; θ_2 is the intercept for the regression on

log(λ); θ_3 is the logarithm of the shape parameter κ in the Weibull distribution; θ_4 and θ_5 are the logarithms of the variances, σ_U^2 and σ_V^2 respectively, of the random effects U and V; θ_6 is $\log\{(1+\phi)/(1-\phi)\}$ where ϕ is the correlation between U and V.

For the model with covariates the correspondence is: θ_1 is the intercept for the regression on the log-odds of prevalence; θ_2 is the slope parameter for the effect of forest cover on the log-odds of prevalence; θ_3 and θ_4 are the slope parameters for the elevation effect on log-odds of prevalence covering the elevation ranges 0-1000 and 1000+, respectively; θ_5 is the slope parameter for the effect of temperature ($\times$ 10) on log-odds of prevalence; θ_6 is the intercept for the regression on log(λ); θ_7 is the slope parameters for the effect of forest cover on log(λ)); θ_8 and θ_9 are the slope parameters for the elevation effect on log(λ) covering the elevation ranges 0-1000 and 1000+, respectively; θ_{10} is the slope parameters for the effect of temperature ($\times$ 10)) on log(λ)); θ_{11} is the logarithm of the shape parameter κ in the Weibull distribution; θ_{12} and θ_{13} are the logarithms of the variances, σ_U^2 and σ_V^2 respectively, of the random effects U and V; θ_{14} is $\log\{(1+\phi)/(1-\phi)\}$ where ϕ is the correlation between U and V.

For the model that uses site as a covariate the correspondence is as follows: θ_1 is the intercept for the regression on the log-odds of prevalence; θ_2 to θ_5 are the differences in effect on the log-odds of prevalence between the study site being DRC North-West, Congo, Cameroon West and Camroon East, respectively, and it being DRC Bas Congo; θ_6 is the intercept for the regression on log(λ); θ_7 to θ_{10} are the differences in effect on log(λ) between the study site being DRC North-West, Congo, Cameroon West and Cameroon East, respectively, and it being DRC Bas Congo; θ_{11} is the logarithm of the shape parameter κ in the Weibull distribution; θ_{12} and θ_{13} are the logarithms of the variances, σ_U^2 and σ_V^2 respectively, of the random effects U and V; θ_{14} is $\log\{(1+\phi)/(1-\phi)\}$ where ϕ is the correlation between U and V.

Estimates and standard errors for the transformed parameters, and 95% confidence intervals for the original parameters, are given in Table A.

S.3 Algorithm to simulate samples from the plug-in predictive distribution

Let Z denote the number of infected individuals out of a sample of size n, Y the vector of infection levels for each of the infected individuals, and (U, V) the bivariate random effect, as defined in equations (3) and (4) (main article). Write $T = \rho(U)\{1 - G(c; V)\}$, suppressing the dependence of T on model parameters and covariates as these are assumed known. We wish to draw samples from the predictive distribution of T given Z and n.

Table A: Parameter estimates for the final model. Estimates and standard errors are for the parameterisation described above. 95% confidence intervals are on the natural scale for each parameter.

Parameter	Estimate	Std.Error	Parameter	95% CI
model without covariates				
θ_1	-2.47	0.125	θ_1	-2.71, -2.22
θ_2	8.2	0.0971	θ_2	8.01, 8.39
θ_3	-0.588	0.0149	κ	0.539, 0.572
θ_4	1.06	0.128	σ_U^2	2.26, 3.72
θ_5	-0.739	0.268	σ_V^2	0.283, 0.807
θ_6	1.91	0.364	ϕ	0.534, 0.864
model with covariates				
θ_1	-2.91	0.219	θ_1	-3.34, -2.48
θ_2	2.27	0.33	θ_2	1.62, 2.91
θ_3	-0.00169	0.000529	θ_3	-0.00263, -0.000657
θ_4	-0.00156	0.000927	θ_4	-0.00338, 0.000258
θ_5	-0.036	0.0146	θ_5	-0.0645, -0.00748
θ_6	7.99	0.172	θ_6	7.65, 8.33
θ_7	0.7	0.236	θ_7	0.237, 1.16
θ_8	-0.000787	0.000341	θ_8	-0.00146, -0.000119
θ_9	-0.000814	0.000644	θ_9	-0.00208, 0.000448
θ_{10}	-0.0254	0.00894	θ_{10}	-0.0429, -0.00787
θ_{11}	-0.588	0.0149	κ	0.539, 0.572
θ_{12}	0.727	0.12	σ_U^2	1.637, 2.616
θ_{13}	-0.967	0.254	σ_V^2	0.231, 0.625
θ_{14}	1.66	0.346	ϕ	0.454, 0.824
model with study site as covariate				
θ_1	-2.75	0.251	θ_1	-3.24, -2.25
θ_2	-0.506	0.334	θ_2	-1.16, 0.149
θ_3	0.733	0.339	θ_3	0.0684, 1.397
θ_4	-0.159	0.348	θ_4	-0.833, 0.514
θ_5	1.86	0.362	θ_5	1.152, 2.57
θ_6	8.1	0.157	θ_6	7.79, 8.41
θ_7	-0.12	0.193	θ_7	-0.497, 0.259
θ_8	0.339	0.182	θ_8	-0.0178, 0.696
θ_9	-0.235	0.188	θ_9	-0.603, 0.133
θ_{10}	0.783	0.192	θ_{10}	0.407, 1.16
θ_{11}	-0.589	0.015	κ	0.539, 0.571
θ_{12}	0.803	0.128	σ_U^2	1.74, 2.87
θ_{13}	-1.19	0.287	σ_V^2	0.173, 0.533
θ_{14}	1.5	0.388	ϕ	0.355, 0.812

In general we can factorise the joint distribution of the random variables U, V, Z and Y as

$$[U,V,Z,Y] = [U][V|U][Z|U,V][Y|U,V,Z]. \tag{1}$$

Integrating both sides of (1) with respect to Y gives

$$[U,V,Z] = [U][V|U][Z|U,V]. \tag{2}$$

For the model defined by equations (2), (3) and (4) (main article), $[Z|U,V] = [Z|U]$, hence

$$[U,V,Z] = [U][Z|U] \times [V|U] \tag{3}$$

Also, $[V|U]$ is a univariate Normal distribution with known mean and variance, $m = \phi U \times \sigma_U/\sigma_V$ and $v = (1-\phi^2)\sigma_V^2$. Hence, the problem reduces to sampling first a value, u say, from the predictive distribution $[U|Z]$ and then sampling from the Normal distribution $[V|u]$. The second of these is straightforward; we used the R function `rnorm()`. For the first, write

$$[U|Z] = [U][Z|U]/[Z]$$

.

The algebraic expressions for each of $[U]$ and $[Z|U]$ are known; the former is a univariate Normal density, the latter a binomial probability with n trials and success probability$\rho(U)$. The marginal distribution of Z, $[Z] = \int_{-\infty}^{+\infty}[U][Z|U]du$, can easily be calculated using Gauss-Hermite quadrature. Thus knowing the probability distribution $[U|Z]$, we can sample from it using the inverse probability integral transform as follows.
First we compute a look-up table for the cumulative distribution $F(U|Z)$ for all the combinations of n and Z observed in the data. For this we generate an equally spaced vector of possible values of U covering the interval $[\mu_u - 3\sigma_u\ ,\ \mu_u + 3\sigma_u]$ with a spacing of 0.1. The cumulative probability $F(U = u_i|Z)$ of each of these values u_i, is approximated by

$$F(u_i|Z) = \begin{cases} 0, & u_i < \mu_u - 3\sigma_u \\ 0.1 \times \sum_{l=1}^{i}[U = \frac{1}{2}(u_{l-1} + u_l)|Z]), & u_i \in [\mu_u - 3\sigma_u, \mu_u + 3\sigma_u] \\ 1, & u_i > \mu_u + 3\sigma_u. \end{cases}$$

Given n and Z for a specific village, a sample from $[U|Z]$ is then generated through the following steps.

1. generate a sample $y_1, ...y_n$ from the unifrom distribution $\mathcal{U}$ (0,1)

2. for each element y_j of this sample, find the largest cumulative probability $F(u_i|Z)$ for the given n and Z such that $y_j \geq F(u_i|Z)$

3. if $y_j = F(u_i|Z)$, the corresponding sample from $[U|Z]$ is u_i,
otherwise the corresponding sample will lie between u_i and u_{i+1} and we estimate it by linearly interpolating the two points and inverting this line at y_j.

Using the above approach we generate 100 000 samples from $[U|Z]$, and in turn from $[V|U]$, which we then use to calculate the sampled values from the plug-in predictive distribution of T.

Table B gives point predictions and 95% predictive intervals for $T = \mathrm{P}(Y > 8000/\text{ml blood}$ for each of the 222 villages.

Table B: Model-based point predictions and 95% prediction intervals (PI) for T, the proportion of individuals with parasite counts greater than 8000/ml blood. Predictions are made for each of the 222 villages for which data are available, using the fitted model without covariates. Point predictions and 95% prediction intervals resulting from a sensitivity analysis (SA) (see main article) are also given .

Village	n	Z	Point prediction	95% PI	Point prediction (SA)	95% PI (SA)
301	49	19	1.19E-01	4.84E-02, 2.23E-01	1.20E-01	4.73E-02, 2.27E-01
302	47	20	1.35E-01	5.63E-02, 2.46E-01	1.35E-01	5.54E-02, 2.48E-01
303	57	25	1.42E-01	6.17E-02, 2.51E-01	1.42E-01	6.10E-02, 2.55E-01
304	68	16	6.29E-02	2.23E-02, 1.30E-01	6.29E-02	2.23E-02, 1.31E-01
305	61	19	9.02E-02	3.52E-02, 1.74E-01	9.00E-02	3.39E-02, 1.74E-01
306	84	22	7.30E-02	2.82E-02, 1.42E-01	7.27E-02	2.81E-02, 1.44E-01
307	53	4	1.43E-02	2.50E-03, 4.80E-02	1.43E-02	2.28E-03, 4.76E-02
308	54	14	7.05E-02	2.49E-02, 1.46E-01	7.02E-02	2.36E-02, 1.47E-01
309	85	17	5.14E-02	1.81E-02, 1.07E-01	5.12E-02	1.78E-02, 1.09E-01
310	78	27	1.05E-01	4.42E-02, 1.91E-01	1.04E-01	4.35E-02, 1.93E-01
311	85	24	8.06E-02	3.20E-02, 1.53E-01	8.05E-02	3.21E-02, 1.53E-01
312	28	6	5.12E-02	1.27E-02, 1.35E-01	5.11E-02	1.22E-02, 1.35E-01
313	82	14	4.18E-02	1.37E-02, 9.20E-02	4.16E-02	1.31E-02, 9.27E-02
314	70	23	9.74E-02	3.96E-02, 1.82E-01	9.73E-02	3.85E-02, 1.83E-01
315	32	9	7.50E-02	2.34E-02, 1.70E-01	7.46E-02	2.32E-02, 1.70E-01
316	100	21	5.53E-02	2.04E-02, 1.11E-01	5.50E-02	2.03E-02, 1.10E-01
317	80	21	7.34E-02	2.86E-02, 1.43E-01	7.28E-02	2.76E-02, 1.45E-01
318	81	18	5.89E-02	2.15E-02, 1.20E-01	5.87E-02	2.08E-02, 1.21E-01
319	57	10	4.23E-02	1.27E-02, 9.97E-02	4.25E-02	1.19E-02, 1.00E-01
320	91	25	7.82E-02	3.13E-02, 1.48E-01	7.84E-02	3.10E-02, 1.49E-01
321	66	22	9.91E-02	4.02E-02, 1.86E-01	9.97E-02	3.99E-02, 1.86E-01
322	84	24	8.19E-02	3.26E-02, 1.56E-01	8.20E-02	3.17E-02, 1.56E-01
323	98	7	1.37E-02	3.14E-03, 3.84E-02	1.38E-02	2.96E-03, 3.88E-02
324	85	11	2.91E-02	8.62E-03, 6.97E-02	2.94E-02	8.41E-03, 7.08E-02
325	58	13	5.83E-02	1.98E-02, 1.26E-01	5.84E-02	1.93E-02, 1.26E-01
327	84	15	4.44E-02	1.50E-02, 9.56E-02	4.39E-02	1.45E-02, 9.66E-02
328	54	9	3.94E-02	1.12E-02, 9.56E-02	3.92E-02	1.09E-02, 9.44E-02
329	56	10	4.32E-02	1.32E-02, 1.02E-01	4.31E-02	1.27E-02, 1.01E-01
331	82	9	2.37E-02	6.37E-03, 6.02E-02	2.37E-02	6.20E-03, 6.07E-02

Continued on next page

Table B–continued from previous page

Village	n	Z	Point prediction	95% PI	Point prediction (SA)	95% PI (SA)
332	75	3	6.65E-03	9.00E-04, 2.62E-02	6.57E-03	8.03E-04, 2.59E-02
333	86	2	3.57E-03	3.37E-04, 1.69E-02	3.59E-03	3.09E-04, 1.74E-02
334	33	0	1.62E-03	3.84E-05, 1.73E-02	1.60E-03	2.97E-05, 1.75E-02
335	57	0	1.01E-03	2.69E-05, 1.03E-02	9.95E-04	2.02E-05, 1.05E-02
336	42	1	4.02E-03	2.27E-04, 2.51E-02	4.06E-03	1.95E-04, 2.44E-02
338	60	1	2.79E-03	1.55E-04, 1.70E-02	2.74E-03	1.28E-04, 1.74E-02
339	55	2	5.99E-03	5.88E-04, 2.75E-02	5.99E-03	5.14E-04, 2.75E-02
340	51	1	3.29E-03	1.87E-04, 2.02E-02	3.37E-03	1.54E-04, 2.02E-02
341	85	6	1.34E-02	2.83E-03, 3.98E-02	1.35E-02	2.85E-03, 4.02E-02
342	54	4	1.38E-02	2.38E-03, 4.66E-02	1.40E-02	2.23E-03, 4.72E-02
343	48	10	5.22E-02	1.60E-02, 1.20E-01	5.22E-02	1.54E-02, 1.21E-01
344	85	2	3.61E-03	3.35E-04, 1.73E-02	3.72E-03	3.17E-04, 1.76E-02
345	73	3	6.85E-03	8.90E-04, 2.68E-02	6.82E-03	8.77E-04, 2.76E-02
346	83	3	5.90E-03	7.83E-04, 2.32E-02	5.93E-03	7.07E-04, 2.39E-02
347	90	20	5.93E-02	2.20E-02, 1.19E-01	5.93E-02	2.11E-02, 1.18E-01
348	60	2	5.37E-03	5.25E-04, 2.54E-02	5.39E-03	4.59E-04, 2.49E-02
349	27	1	6.38E-03	3.55E-04, 3.93E-02	6.22E-03	2.91E-04, 3.88E-02
350	60	2	5.41E-03	5.20E-04, 2.53E-02	5.38E-03	4.97E-04, 2.48E-02
351	90	6	1.25E-02	2.62E-03, 3.74E-02	1.26E-02	2.59E-03, 3.88E-02
352	50	6	2.54E-02	5.90E-03, 7.20E-02	2.58E-02	5.73E-03, 7.16E-02
353	50	4	1.51E-02	2.63E-03, 5.09E-02	1.51E-02	2.52E-03, 5.09E-02
201	81	14	4.24E-02	1.43E-02, 9.32E-02	4.21E-02	1.35E-02, 9.20E-02
202	80	7	1.76E-02	4.14E-03, 4.87E-02	1.75E-02	4.06E-03, 4.95E-02
203	84	29	1.05E-01	4.45E-02, 1.89E-01	1.05E-01	4.25E-02, 1.90E-01
204	82	16	4.97E-02	1.72E-02, 1.04E-01	5.00E-02	1.68E-02, 1.06E-01
205	81	31	1.21E-01	5.22E-02, 2.13E-01	1.21E-01	5.16E-02, 2.12E-01
206	82	25	8.87E-02	3.59E-02, 1.66E-01	8.93E-02	3.56E-02, 1.69E-01
207	86	29	1.02E-01	4.28E-02, 1.84E-01	1.02E-01	4.23E-02, 1.84E-01
208	82	19	6.21E-02	2.29E-02, 1.25E-01	6.17E-02	2.30E-02, 1.28E-01
209	85	20	6.35E-02	2.38E-02, 1.26E-01	6.38E-02	2.32E-02, 1.28E-01
210	81	27	1.00E-01	4.15E-02, 1.83E-01	9.98E-02	4.06E-02, 1.84E-01
211	80	22	7.77E-02	3.03E-02, 1.49E-01	7.69E-02	2.99E-02, 1.48E-01
212	80	18	5.98E-02	2.19E-02, 1.22E-01	5.98E-02	2.13E-02, 1.23E-01
213	80	28	1.07E-01	4.50E-02, 1.93E-01	1.07E-01	4.32E-02, 1.98E-01
214	81	27	1.00E-01	4.14E-02, 1.82E-01	1.01E-01	4.09E-02, 1.82E-01
215	80	23	8.24E-02	3.26E-02, 1.57E-01	8.23E-02	3.16E-02, 1.59E-01
216	69	1	2.38E-03	1.35E-04, 1.48E-02	2.41E-03	1.27E-04, 1.49E-02
217	93	0	6.34E-04	2.03E-05, 6.31E-03	6.45E-04	1.34E-05, 6.37E-03
218	96	0	6.19E-04	2.01E-05, 5.99E-03	6.54E-04	1.33E-05, 6.35E-03
219	88	2	3.47E-03	3.33E-04, 1.66E-02	3.50E-03	3.05E-04, 1.68E-02
220	78	0	7.53E-04	2.23E-05, 7.62E-03	7.49E-04	1.56E-05, 7.72E-03

Continued on next page

Table B–continued from previous page

Village	n	Z	Point prediction	95% PI	Point prediction (SA)	95% PI (SA)
221	84	0	7.10E-04	2.17E-05, 7.03E-03	7.15E-04	1.53E-05, 6.89E-03
222	89	0	6.72E-04	2.12E-05, 6.55E-03	6.79E-04	1.44E-05, 6.74E-03
223	85	0	6.99E-04	2.16E-05, 6.90E-03	7.07E-04	1.63E-05, 6.85E-03
224	85	0	7.07E-04	2.23E-05, 7.01E-03	7.04E-04	1.66E-05, 7.22E-03
225	81	1	2.03E-03	1.15E-04, 1.25E-02	2.02E-03	9.53E-05, 1.31E-02
226	86	0	6.99E-04	2.15E-05, 6.81E-03	6.98E-04	1.62E-05, 6.90E-03
227	84	0	7.13E-04	2.28E-05, 7.07E-03	7.02E-04	1.59E-05, 7.26E-03
228	87	1	1.87E-03	1.08E-04, 1.16E-02	1.86E-03	9.29E-05, 1.18E-02
229	60	0	9.66E-04	2.81E-05, 9.75E-03	9.65E-04	2.09E-05, 1.02E-02
230	84	0	7.05E-04	2.19E-05, 6.93E-03	7.17E-04	1.28E-05, 7.04E-03
231	81	3	6.08E-03	7.93E-04, 2.40E-02	6.05E-03	7.09E-04, 2.39E-02
232	80	3	6.12E-03	8.06E-04, 2.43E-02	6.18E-03	7.67E-04, 2.46E-02
233	80	0	7.41E-04	2.24E-05, 7.37E-03	7.50E-04	1.59E-05, 7.56E-03
234	80	0	7.43E-04	2.27E-05, 7.41E-03	7.43E-04	1.74E-05, 7.20E-03
235	80	3	6.14E-03	8.02E-04, 2.44E-02	6.12E-03	7.78E-04, 2.37E-02
236	81	6	1.42E-02	3.07E-03, 4.22E-02	1.43E-02	2.71E-03, 4.27E-02
237	81	1	2.00E-03	1.16E-04, 1.24E-02	2.01E-03	9.20E-05, 1.25E-02
238	81	3	6.05E-03	8.18E-04, 2.41E-02	6.09E-03	7.36E-04, 2.42E-02
239	80	4	8.68E-03	1.43E-03, 3.04E-02	8.65E-03	1.42E-03, 3.08E-02
240	80	20	6.87E-02	2.59E-02, 1.36E-01	6.83E-02	2.55E-02, 1.37E-01
241	85	0	6.96E-04	2.24E-05, 6.89E-03	7.13E-04	1.72E-05, 6.93E-03
242	46	0	1.21E-03	3.15E-05, 1.25E-02	1.27E-03	2.47E-05, 1.32E-02
243	82	0	7.24E-04	2.17E-05, 7.25E-03	7.45E-04	1.63E-05, 7.22E-03
244	85	7	1.63E-02	3.82E-03, 4.57E-02	1.65E-02	3.71E-03, 4.64E-02
245	85	3	5.67E-03	7.46E-04, 2.26E-02	5.75E-03	7.18E-04, 2.30E-02
246	89	4	7.63E-03	1.22E-03, 2.68E-02	7.69E-03	1.14E-03, 2.73E-02
247	85	5	1.06E-02	2.04E-03, 3.41E-02	1.09E-02	1.88E-03, 3.37E-02
248	89	2	3.45E-03	3.25E-04, 1.64E-02	3.50E-03	2.94E-04, 1.61E-02
249	80	0	7.45E-04	2.20E-05, 7.30E-03	7.54E-04	1.66E-05, 7.43E-03
250	87	0	6.87E-04	2.08E-05, 6.71E-03	7.03E-04	1.59E-05, 7.11E-03
101	79	16	5.21E-02	1.81E-02, 1.10E-01	5.23E-02	1.71E-02, 1.11E-01
102	79	19	6.51E-02	2.43E-02, 1.31E-01	6.50E-02	2.38E-02, 1.29E-01
103	80	16	5.13E-02	1.80E-02, 1.08E-01	5.09E-02	1.75E-02, 1.08E-01
104	81	20	6.77E-02	2.56E-02, 1.34E-01	6.77E-02	2.47E-02, 1.34E-01
105	78	17	5.73E-02	2.05E-02, 1.18E-01	5.70E-02	2.02E-02, 1.18E-01
106	76	29	1.19E-01	5.11E-02, 2.12E-01	1.19E-01	5.07E-02, 2.14E-01
107	83	20	6.55E-02	2.48E-02, 1.30E-01	6.54E-02	2.43E-02, 1.32E-01
108	82	31	1.18E-01	5.12E-02, 2.09E-01	1.18E-01	5.11E-02, 2.11E-01
109	64	14	5.71E-02	1.95E-02, 1.21E-01	5.66E-02	1.90E-02, 1.20E-01
110	76	21	7.79E-02	3.06E-02, 1.51E-01	7.91E-02	3.06E-02, 1.53E-01
111	88	15	4.19E-02	1.40E-02, 9.09E-02	4.19E-02	1.35E-02, 9.10E-02

Continued on next page

Table B–continued from previous page

Village	n	Z	Point prediction	95% PI	Point prediction (SA)	95% PI (SA)
112	88	18	5.32E-02	1.92E-02, 1.10E-01	5.30E-02	1.84E-02, 1.11E-01
113	93	21	6.05E-02	2.26E-02, 1.20E-01	6.04E-02	2.15E-02, 1.22E-01
114	83	21	6.99E-02	2.68E-02, 1.37E-01	7.00E-02	2.62E-02, 1.37E-01
115	85	0	6.98E-04	2.15E-05, 6.95E-03	7.18E-04	1.53E-05, 7.29E-03
116	102	0	5.86E-04	1.94E-05, 5.75E-03	5.82E-04	1.38E-05, 5.68E-03
117	89	5	1.00E-02	1.91E-03, 3.21E-02	1.01E-02	1.72E-03, 3.21E-02
118	47	3	1.14E-02	1.58E-03, 4.34E-02	1.11E-02	1.36E-03, 4.37E-02
119	85	14	4.00E-02	1.31E-02, 8.83E-02	4.00E-02	1.24E-02, 8.70E-02
120	81	5	1.13E-02	2.16E-03, 3.59E-02	1.14E-02	2.03E-03, 3.60E-02
121	94	0	6.34E-04	2.02E-05, 6.21E-03	6.60E-04	1.36E-05, 6.35E-03
122	81	0	7.23E-04	2.26E-05, 7.16E-03	7.58E-04	1.72E-05, 7.69E-03
123	99	0	6.11E-04	1.97E-05, 5.97E-03	6.12E-04	1.44E-05, 6.17E-03
124	88	2	3.49E-03	3.32E-04, 1.64E-02	3.50E-03	3.02E-04, 1.66E-02
125	89	0	6.77E-04	2.11E-05, 6.56E-03	6.72E-04	1.37E-05, 6.52E-03
126	85	0	7.01E-04	2.18E-05, 6.83E-03	6.88E-04	1.46E-05, 6.67E-03
127	85	0	6.98E-04	2.16E-05, 6.85E-03	7.00E-04	1.68E-05, 6.95E-03
128	80	0	7.50E-04	2.25E-05, 7.45E-03	7.57E-04	1.54E-05, 7.54E-03
129	85	3	5.71E-03	7.50E-04, 2.28E-02	5.86E-03	6.78E-04, 2.34E-02
130	95	9	1.97E-02	5.19E-03, 5.08E-02	1.98E-02	5.03E-03, 5.14E-02
131	81	0	7.30E-04	2.22E-05, 7.27E-03	7.53E-04	1.61E-05, 7.30E-03
132	81	0	7.37E-04	2.20E-05, 7.21E-03	7.45E-04	1.66E-05, 7.50E-03
133	73	0	8.07E-04	2.39E-05, 8.08E-03	8.11E-04	1.53E-05, 8.14E-03
134	81	2	3.84E-03	3.76E-04, 1.81E-02	3.84E-03	3.24E-04, 1.85E-02
135	82	1	1.97E-03	1.10E-04, 1.24E-02	2.02E-03	1.06E-04, 1.21E-02
136	81	1	2.01E-03	1.11E-04, 1.26E-02	2.05E-03	9.74E-05, 1.34E-02
137	81	0	7.27E-04	2.23E-05, 7.29E-03	7.42E-04	1.62E-05, 7.27E-03
138	80	1	2.03E-03	1.14E-04, 1.27E-02	2.09E-03	9.86E-05, 1.27E-02
139	82	11	3.07E-02	9.15E-03, 7.26E-02	3.06E-02	9.17E-03, 7.40E-02
140	82	30	1.13E-01	4.87E-02, 2.02E-01	1.13E-01	4.76E-02, 2.03E-01
141	82	23	7.99E-02	3.16E-02, 1.53E-01	8.03E-02	3.05E-02, 1.52E-01
142	82	15	4.56E-02	1.54E-02, 9.86E-02	4.57E-02	1.46E-02, 9.83E-02
143	80	16	5.13E-02	1.78E-02, 1.08E-01	5.08E-02	1.69E-02, 1.07E-01
144	81	7	1.74E-02	4.10E-03, 4.82E-02	1.72E-02	3.79E-03, 4.87E-02
145	81	5	1.13E-02	2.19E-03, 3.59E-02	1.13E-02	2.00E-03, 3.59E-02
146	81	11	3.10E-02	9.16E-03, 7.36E-02	3.12E-02	8.87E-03, 7.44E-02
147	80	13	3.92E-02	1.26E-02, 8.76E-02	3.88E-02	1.21E-02, 8.79E-02
148	57	10	4.23E-02	1.27E-02, 9.95E-02	4.25E-02	1.21E-02, 9.95E-02
149	81	23	8.11E-02	3.20E-02, 1.55E-01	8.14E-02	3.19E-02, 1.57E-01
703	45	1	3.78E-03	2.14E-04, 2.31E-02	3.73E-03	2.05E-04, 2.37E-02
704	57	4	1.30E-02	2.20E-03, 4.39E-02	1.30E-02	2.01E-03, 4.37E-02
705	134	11	1.66E-02	4.60E-03, 4.13E-02	1.66E-02	4.41E-03, 4.17E-02

Continued on next page

Table B–continued from previous page

Village	n	Z	Point prediction	95% PI	Point prediction (SA)	95% PI (SA)
706	121	7	1.06E-02	2.36E-03, 3.06E-02	1.06E-02	2.17E-03, 3.14E-02
707	158	10	1.19E-02	3.07E-03, 3.14E-02	1.20E-02	2.90E-03, 3.22E-02
708	128	8	1.17E-02	2.79E-03, 3.24E-02	1.17E-02	2.74E-03, 3.25E-02
709	174	15	1.79E-02	5.30E-03, 4.18E-02	1.81E-02	5.13E-03, 4.28E-02
710	107	18	4.15E-02	1.43E-02, 8.75E-02	4.12E-02	1.42E-02, 8.91E-02
711	181	20	2.46E-02	8.05E-03, 5.34E-02	2.48E-02	7.68E-03, 5.37E-02
712	30	4	2.78E-02	5.16E-03, 8.87E-02	2.75E-02	5.06E-03, 8.89E-02
714	26	4	3.28E-02	6.05E-03, 1.03E-01	3.31E-02	5.97E-03, 1.03E-01
715	39	3	1.41E-02	2.00E-03, 5.30E-02	1.43E-02	1.91E-03, 5.37E-02
716	66	3	7.72E-03	1.04E-03, 2.98E-02	7.74E-03	9.49E-04, 2.97E-02
717	24	1	7.08E-03	3.81E-04, 4.42E-02	7.10E-03	3.54E-04, 4.64E-02
718	88	5	1.03E-02	1.94E-03, 3.28E-02	1.01E-02	1.79E-03, 3.31E-02
720	195	0	3.14E-04	1.28E-05, 2.97E-03	3.22E-04	8.07E-06, 3.00E-03
721	125	4	5.10E-03	7.85E-04, 1.84E-02	5.06E-03	7.27E-04, 1.84E-02
722	115	3	3.97E-03	5.07E-04, 1.63E-02	4.02E-03	4.52E-04, 1.62E-02
723	146	1	1.05E-03	5.99E-05, 6.60E-03	1.06E-03	5.09E-05, 6.81E-03
724	78	1	2.11E-03	1.19E-04, 1.30E-02	2.09E-03	9.61E-05, 1.32E-02
725	68	5	1.40E-02	2.75E-03, 4.38E-02	1.42E-02	2.57E-03, 4.45E-02
726	119	3	3.82E-03	4.91E-04, 1.55E-02	3.85E-03	3.93E-04, 1.59E-02
727	94	3	5.06E-03	6.57E-04, 2.02E-02	5.09E-03	5.76E-04, 2.06E-02
728	91	46	1.76E-01	8.31E-02, 2.86E-01	1.76E-01	8.25E-02, 2.89E-01
729	112	49	1.45E-01	6.75E-02, 2.42E-01	1.46E-01	6.69E-02, 2.44E-01
730	96	51	1.88E-01	9.16E-02, 3.03E-01	1.87E-01	8.85E-02, 3.06E-01
731	70	29	1.33E-01	5.86E-02, 2.33E-01	1.33E-01	5.64E-02, 2.37E-01
732	52	18	1.03E-01	4.05E-02, 1.97E-01	1.03E-01	3.94E-02, 2.00E-01
733	229	24	2.33E-02	7.75E-03, 4.93E-02	2.31E-02	7.40E-03, 4.99E-02
734	111	11	2.11E-02	5.97E-03, 5.16E-02	2.10E-02	5.55E-03, 5.19E-02
735	66	7	2.22E-02	5.34E-03, 6.08E-02	2.25E-02	5.18E-03, 6.08E-02
736	107	48	1.50E-01	7.00E-02, 2.48E-01	1.50E-01	6.91E-02, 2.51E-01
737	95	31	9.82E-02	4.14E-02, 1.78E-01	9.84E-02	4.03E-02, 1.79E-01
739	151	0	4.02E-04	1.52E-05, 3.83E-03	4.22E-04	9.29E-06, 3.96E-03
740	140	0	4.37E-04	1.55E-05, 4.16E-03	4.36E-04	1.05E-05, 4.42E-03
741	52	0	1.10E-03	2.96E-05, 1.12E-02	1.08E-03	2.23E-05, 1.12E-02
742	186	0	3.26E-04	1.27E-05, 3.08E-03	3.32E-04	8.36E-06, 3.15E-03
743	126	0	4.79E-04	1.68E-05, 4.66E-03	4.99E-04	1.06E-05, 5.09E-03
744	105	0	5.73E-04	1.93E-05, 5.59E-03	5.74E-04	1.33E-05, 5.95E-03
745	133	3	3.35E-03	4.27E-04, 1.38E-02	3.42E-03	3.58E-04, 1.42E-02
746	212	0	2.87E-04	1.18E-05, 2.68E-03	2.95E-04	8.00E-06, 2.75E-03
901	118	26	5.91E-02	2.26E-02, 1.15E-01	5.90E-02	2.17E-02, 1.15E-01
902	127	37	8.52E-02	3.56E-02, 1.54E-01	8.58E-02	3.48E-02, 1.55E-01
903	123	32	7.37E-02	2.98E-02, 1.36E-01	7.42E-02	2.87E-02, 1.38E-01

Continued on next page

Table B–continued from previous page

Village	n	Z	Point prediction	95% PI	Point prediction (SA)	95% PI (SA)
905	108	29	7.65E-02	3.06E-02, 1.42E-01	7.70E-02	2.97E-02, 1.44E-01
906	119	26	5.87E-02	2.26E-02, 1.14E-01	5.85E-02	2.24E-02, 1.15E-01
907	107	41	1.22E-01	5.43E-02, 2.09E-01	1.22E-01	5.34E-02, 2.12E-01
911	99	37	1.17E-01	5.14E-02, 2.04E-01	1.18E-01	5.05E-02, 2.08E-01
912	83	22	7.44E-02	2.86E-02, 1.43E-01	7.42E-02	2.80E-02, 1.45E-01
913	97	32	9.95E-02	4.26E-02, 1.79E-01	9.93E-02	4.08E-02, 1.79E-01
914	148	56	1.21E-01	5.54E-02, 2.03E-01	1.21E-01	5.37E-02, 2.06E-01
915	25	9	1.02E-01	3.37E-02, 2.20E-01	1.02E-01	3.26E-02, 2.27E-01
916	89	13	3.42E-02	1.07E-02, 7.78E-02	3.39E-02	1.03E-02, 7.59E-02
917	41	16	1.19E-01	4.70E-02, 2.29E-01	1.19E-01	4.66E-02, 2.29E-01
918	143	17	2.69E-02	8.67E-03, 5.94E-02	2.66E-02	8.52E-03, 5.95E-02
919	109	31	8.24E-02	3.35E-02, 1.52E-01	8.25E-02	3.29E-02, 1.52E-01
920	130	53	1.33E-01	6.11E-02, 2.23E-01	1.33E-01	6.05E-02, 2.25E-01
922	99	29	8.52E-02	3.46E-02, 1.58E-01	8.55E-02	3.49E-02, 1.59E-01
923	110	41	1.17E-01	5.22E-02, 2.02E-01	1.18E-01	5.09E-02, 2.05E-01
924	110	26	6.46E-02	2.52E-02, 1.24E-01	6.45E-02	2.47E-02, 1.24E-01
925	109	38	1.08E-01	4.66E-02, 1.88E-01	1.07E-01	4.63E-02, 1.88E-01
926	25	11	1.34E-01	4.91E-02, 2.69E-01	1.35E-01	4.71E-02, 2.68E-01
927	69	22	9.36E-02	3.76E-02, 1.76E-01	9.42E-02	3.73E-02, 1.78E-01
928	125	47	1.19E-01	5.34E-02, 2.03E-01	1.19E-01	5.13E-02, 2.04E-01
931	128	28	5.90E-02	2.29E-02, 1.14E-01	5.88E-02	2.18E-02, 1.14E-01
933	87	24	7.85E-02	3.09E-02, 1.49E-01	7.80E-02	2.96E-02, 1.51E-01
936	107	26	6.69E-02	2.60E-02, 1.28E-01	6.81E-02	2.65E-02, 1.30E-01
937	78	20	7.10E-02	2.69E-02, 1.39E-01	7.12E-02	2.67E-02, 1.40E-01
938	110	9	1.63E-02	4.23E-03, 4.29E-02	1.64E-02	4.22E-03, 4.28E-02
939	107	38	1.10E-01	4.81E-02, 1.93E-01	1.10E-01	4.69E-02, 1.95E-01
940	82	33	1.29E-01	5.70E-02, 2.23E-01	1.30E-01	5.60E-02, 2.27E-01
941	93	44	1.60E-01	7.49E-02, 2.65E-01	1.59E-01	7.50E-02, 2.67E-01
950	69	32	1.55E-01	7.02E-02, 2.63E-01	1.54E-01	6.73E-02, 2.63E-01

S.4 Data-analysis to support exclusion of the available environmental covariates from the model.

Environmental covariate effects were statistically significant, but their inclusion made very little difference to predictions of the proportions of highly infected individuals in each village (Figure C).

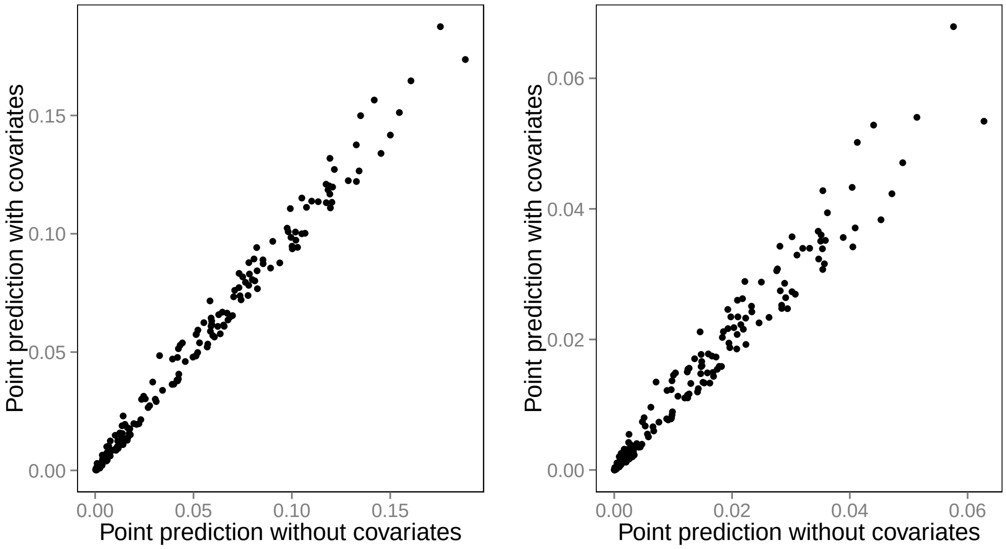

Figure C: Predicted proportions of highly infected individuals in the model that includes no environmental covariates against the corresponding predictions in the model that includes forest cover, elevation and temperature. In the left-hand panel, the threshold for high infection is $c = 8000$, in the right-hand panel the threshold is $c = 30\,000$.

S.5 Data-analysis to show that site differences make a very small contribution to the variability between villages.

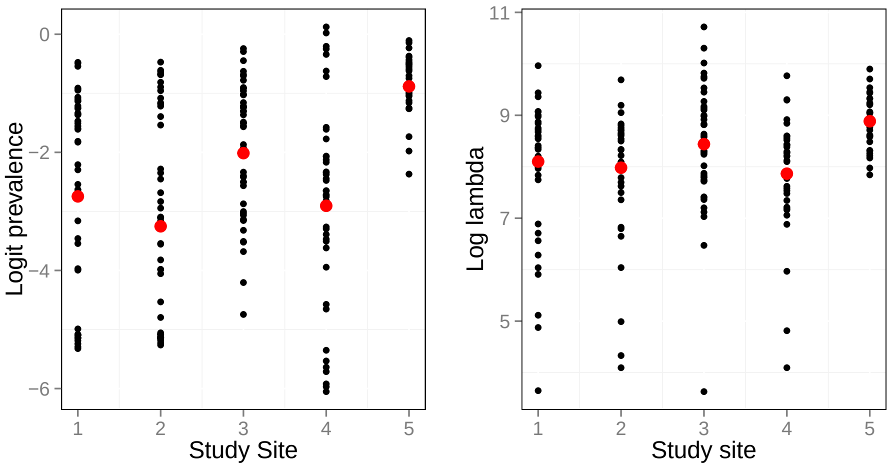

Figure D: Empirical log-odds of the prevalence and log lambda plotted against study site. Red dots show the fitted coefficients for each study-site in the logistic linear model for prevalence (left-hand panel) and in the log-linear model for intensity of infection (right-hand panel).

S.6 Model Validation

Although desirable, formal model validation is not possible as the target of prediction, the proportion of individuals in a village, which exceeds infection levels of a certain threshold, cannot be observed unless all individuals in a community are tested and their blood samples analysed. However, in order to get a sense of the accuracy of out-of-sample predictions, we predict the proportions of highly infected individuals in communities and compare these with the observed proportion of highly infected individuals in the samples and their 95% confidence intervals based on the binomial sampling distribution for datasets from Cameroon, Gabon and Equatorial Guinea that have not been used in the model development. The following tables and figures show the results of this.

Cameroon

Table C: Model-based 95% prediction intervals (PI) for T, the proportion of individuals with parasite counts greater than 8000/ml blood and the proportion of individuals with parasite counts greater than 30000/ml blood as well as the 95% confidence intervals for the true proportions based on the binomial sampling distribution of the observed numbers in each village. n, the number of individuals tested, Z, the number of individuals tested positive for *Loa Loa*, Prop_{8k} is the observed proportion with infection levels greater than 8000mf/ml blood, Prop_{30k} is the observed proportion with infection levels greater than 30000mf/ml blood

Village	n	Z	Prop_{8k}	95% PI_{8k}	95% CI_{8k}	Prop_{30k}	95% PI_{30k}	95% CI_{30k}
NA	24	7	4.20E-02	2.15E-02, 1.82E-01	9.00E-03, 1.67E-01	0.00E+00	2.53E-03, 7.80E-02	0.00E+00, 1.01E-01
NA	25	11	2.00E-01	4.92E-02, 2.68E-01	1.01E-01, 3.58E-01	8.00E-02	7.10E-03, 1.26E-01	2.70E-02, 2.15E-01
NA	27	7	1.11E-01	1.85E-02, 1.62E-01	4.50E-02, 2.48E-01	3.70E-02	2.03E-03, 6.69E-02	8.00E-03, 1.50E-01
NA	29	14	6.90E-02	6.16E-02, 2.90E-01	2.30E-02, 1.88E-01	3.40E-02	9.31E-03, 1.40E-01	8.00E-03, 1.41E-01
NA	40	17	2.50E-02	5.43E-02, 2.49E-01	6.00E-03, 1.05E-01	0.00E+00	7.69E-03, 1.17E-01	0.00E+00, 6.30E-02
NA	51	45	0.00E+00	2.27E-01, 5.59E-01	0.00E+00, 5.00E-02	0.00E+00	5.76E-02, 3.41E-01	0.00E+00, 5.00E-02
NA	59	10	1.02E-01	1.21E-02, 9.55E-02	5.40E-02, 1.85E-01	1.70E-02	1.10E-03, 3.71E-02	4.00E-03, 7.20E-02
NA	62	20	8.10E-02	3.77E-02, 1.80E-01	4.00E-02, 1.57E-01	0.00E+00	4.60E-03, 8.03E-02	0.00E+00, 4.20E-02
NA	64	30	1.56E-01	7.02E-02, 2.68E-01	9.60E-02, 2.45E-01	3.10E-02	1.08E-02, 1.30E-01	1.00E-02, 9.00E-02
NA	65	17	6.20E-02	2.65E-02, 1.45E-01	2.80E-02, 1.30E-01	0.00E+00	3.03E-03, 6.15E-02	0.00E+00, 4.00E-02
4408	73	5	4.10E-02	2.51E-03, 4.02E-02	1.70E-02, 9.80E-02	2.70E-02	1.41E-04, 1.31E-02	9.00E-03, 7.90E-02
NA	77	16	3.90E-02	1.88E-02, 1.12E-01	1.60E-02, 9.40E-02	0.00E+00	1.89E-03, 4.58E-02	0.00E+00, 3.40E-02
NA	77	21	0.00E+00	2.98E-02, 1.49E-01	0.00E+00, 3.40E-02	0.00E+00	3.41E-03, 6.41E-02	0.00E+00, 3.40E-02
4404	80	14	3.80E-02	1.42E-02, 9.52E-02	1.50E-02, 9.00E-02	0.00E+00	1.33E-03, 3.73E-02	0.00E+00, 3.30E-02
NA	82	20	8.50E-02	2.53E-02, 1.32E-01	4.70E-02, 1.50E-01	4.90E-02	2.70E-03, 5.57E-02	2.20E-02, 1.04E-01
NA	83	30	7.20E-02	4.79E-02, 2.00E-01	3.80E-02, 1.34E-01	1.20E-02	6.32E-03, 9.20E-02	3.00E-03, 5.20E-02
NA	85	32	1.18E-01	5.15E-02, 2.08E-01	7.20E-02, 1.87E-01	1.20E-02	6.94E-03, 9.64E-02	3.00E-03, 5.10E-02
NA	88	38	0.00E+00	6.41E-02, 2.41E-01	0.00E+00, 3.00E-02	0.00E+00	9.42E-03, 1.16E-01	0.00E+00, 3.00E-02
4402	89	6	3.40E-02	2.68E-03, 3.77E-02	1.40E-02, 8.10E-02	1.10E-02	1.55E-04, 1.23E-02	3.00E-03, 4.90E-02
NA	92	23	6.50E-02	2.67E-02, 1.34E-01	3.40E-02, 1.21E-01	0.00E+00	2.86E-03, 5.71E-02	0.00E+00, 2.90E-02
NA	93	65	3.20E-02	1.48E-01, 4.18E-01	1.30E-02, 7.80E-02	1.10E-02	2.95E-02, 2.29E-01	2.00E-03, 4.70E-02
4407	94	3	0.00E+00	6.70E-04, 2.05E-02	0.00E+00, 2.80E-02	0.00E+00	2.56E-05, 5.64E-03	0.00E+00, 2.80E-02

Continued on next page

Table C–continued from previous page

Village	n	Z	Prop_{8k}	95% PI_{8k}	95% CI_{8k}	Prop_{30k}	95% PI_{30k}	95% CI_{30k}
NA	96	34	5.20E-02	4.69E-02, 1.93E-01	2.60E-02, 1.03E-01	2.10E-02	6.07E-03, 8.90E-02	7.00E-03, 6.10E-02
NA	100	31	1.20E-01	3.83E-02, 1.66E-01	7.60E-02, 1.84E-01	3.00E-02	4.69E-03, 7.49E-02	1.20E-02, 7.30E-02
NA	109	24	9.20E-02	2.23E-02, 1.16E-01	5.60E-02, 1.48E-01	9.00E-03	2.31E-03, 4.78E-02	2.00E-03, 4.00E-02
NA	109	51	0.00E+00	7.52E-02, 2.60E-01	0.00E+00, 2.40E-02	0.00E+00	1.12E-02, 1.28E-01	0.00E+00, 2.40E-02
4401	111	28	7.20E-02	2.79E-02, 1.33E-01	4.10E-02, 1.23E-01	2.70E-02	3.10E-03, 5.70E-02	1.10E-02, 6.60E-02
NA	118	51	0.00E+00	6.64E-02, 2.37E-01	0.00E+00, 2.20E-02	0.00E+00	9.69E-03, 1.15E-01	0.00E+00, 2.20E-02
NA	119	35	7.60E-02	3.62E-02, 1.56E-01	4.50E-02, 1.26E-01	8.00E-03	4.27E-03, 6.89E-02	2.00E-03, 3.70E-02
NA	121	49	8.00E-03	6.01E-02, 2.21E-01	2.00E-03, 3.60E-02	0.00E+00	8.43E-03, 1.05E-01	0.00E+00, 2.20E-02
NA	121	82	0.00E+00	1.43E-01, 4.01E-01	0.00E+00, 2.20E-02	0.00E+00	2.75E-02, 2.16E-01	0.00E+00, 2.20E-02
NA	124	35	7.30E-02	3.39E-02, 1.48E-01	4.30E-02, 1.21E-01	4.00E-02	3.96E-03, 6.57E-02	2.00E-02, 8.10E-02
4406	125	15	4.00E-02	8.52E-03, 6.10E-02	2.00E-02, 8.00E-02	0.00E+00	6.57E-04, 2.25E-02	0.00E+00, 2.10E-02
NA	125	24	1.04E-01	1.85E-02, 9.89E-02	6.70E-02, 1.58E-01	5.60E-02	1.80E-03, 4.02E-02	3.10E-02, 1.00E-01
4405	130	10	8.00E-03	4.02E-03, 3.92E-02	2.00E-03, 3.40E-02	0.00E+00	2.48E-04, 1.31E-02	0.00E+00, 2.00E-02
NA	132	31	7.60E-02	2.55E-02, 1.22E-01	4.60E-02, 1.23E-01	2.30E-02	2.67E-03, 5.16E-02	9.00E-03, 5.60E-02
NA	137	32	5.80E-02	2.55E-02, 1.21E-01	3.30E-02, 1.01E-01	0.00E+00	2.70E-03, 5.10E-02	0.00E+00, 1.90E-02
4403	140	2	0.00E+00	1.81E-04, 9.84E-03	0.00E+00, 1.90E-02	0.00E+00	4.57E-06, 2.41E-03	0.00E+00, 1.90E-02
NA	140	27	4.30E-02	1.89E-02, 9.78E-02	2.20E-02, 8.10E-02	1.40E-02	1.84E-03, 4.01E-02	5.00E-03, 4.20E-02
NA	154	27	1.90E-02	1.63E-02, 8.78E-02	8.00E-03, 4.80E-02	0.00E+00	1.54E-03, 3.52E-02	0.00E+00, 1.70E-02
NA	156	47	8.30E-02	3.85E-02, 1.58E-01	5.40E-02, 1.27E-01	2.60E-02	4.64E-03, 7.06E-02	1.20E-02, 5.60E-02
NA	158	39	1.01E-01	2.81E-02, 1.27E-01	6.80E-02, 1.48E-01	3.80E-02	3.10E-03, 5.43E-02	2.00E-02, 7.20E-02
NA	172	47	7.60E-02	3.37E-02, 1.41E-01	4.90E-02, 1.16E-01	2.30E-02	3.82E-03, 6.22E-02	1.00E-02, 5.10E-02
NA	178	51	1.29E-01	3.60E-02, 1.48E-01	9.30E-02, 1.76E-01	4.50E-02	4.20E-03, 6.56E-02	2.60E-02, 7.80E-02
NA	180	18	2.80E-02	6.86E-03, 4.85E-02	1.40E-02, 5.60E-02	6.00E-03	4.92E-04, 1.72E-02	1.00E-03, 2.50E-02
NA	180	23	4.40E-02	1.03E-02, 6.21E-02	2.50E-02, 7.70E-02	2.20E-02	8.24E-04, 2.34E-02	1.00E-02, 4.90E-02
NA	192	56	1.41E-01	3.73E-02, 1.50E-01	1.04E-01, 1.87E-01	3.60E-02	4.45E-03, 6.72E-02	2.00E-02, 6.60E-02
NA	234	48	3.80E-02	2.22E-02, 1.01E-01	2.30E-02, 6.50E-02	1.70E-02	2.15E-03, 4.23E-02	8.00E-03, 3.80E-02
NA	443	277	9.00E-03	1.29E-01, 3.57E-01	4.00E-03, 2.00E-02	5.00E-03	2.39E-02, 1.91E-01	1.00E-03, 1.40E-02
				,	,		,	,

Gabon

Table D: Model-based 95% prediction intervals (PI) for T, the proportion of individuals with parasite counts greater than 8000/ml blood the proportion of individuals with parasite counts greater than 30000/ml blood as well as the 95% confidence intervals for the true proportions based on the binomial sampling distribution of the observed numbers in each village. n, the number of individuals tested, Z, the number of individuals tested positive for *Loa Loa*, Prop_{8k} is the observed proportion with infection levels greater than 8000mf/ml blood, Prop_{30k} is the observed proportion with infection levels greater than 30000mf/ml blood

Village	n	Z	Prop_{8k}	95% PI_{8k}	95% CI_{8k}	Prop_{30k}	95% PI_{30k}	95% CI_{30k}
9035	5	1	0.00E+00	1.45E-03, 1.83E-01	0.00E+00, 3.51E-01	0.00E+00	9.65E-05, 7.19E-02	0.00E+00, 3.51E-01
6268	8	1	0.00E+00	9.88E-04, 1.24E-01	0.00E+00, 2.53E-01	0.00E+00	5.84E-05, 4.49E-02	0.00E+00, 2.53E-01
9067	9	3	1.11E-01	1.21E-02, 2.35E-01	2.50E-02, 3.77E-01	1.11E-01	1.31E-03, 1.00E-01	2.50E-02, 3.77E-01
9044	11	4	1.82E-01	1.84E-02, 2.45E-01	6.20E-02, 4.27E-01	0.00E+00	2.30E-03, 1.08E-01	0.00E+00, 1.97E-01
9065	13	1	0.00E+00	6.55E-04, 7.97E-02	0.00E+00, 1.72E-01	0.00E+00	3.24E-05, 2.66E-02	0.00E+00, 1.72E-01
6228	13	3	0.00E+00	7.63E-03, 1.65E-01	0.00E+00, 1.72E-01	0.00E+00	7.38E-04, 6.57E-02	0.00E+00, 1.72E-01
9047	13	3	0.00E+00	7.56E-03, 1.66E-01	0.00E+00, 1.72E-01	0.00E+00	7.41E-04, 6.64E-02	0.00E+00, 1.72E-01
9075	14	2	0.00E+00	2.72E-03, 1.14E-01	0.00E+00, 1.62E-01	0.00E+00	1.97E-04, 4.13E-02	0.00E+00, 1.62E-01
9059	16	1	0.00E+00	5.65E-04, 6.63E-02	0.00E+00, 1.45E-01	0.00E+00	2.55E-05, 2.16E-02	0.00E+00, 1.45E-01
9108	18	9	3.33E-01	5.45E-02, 3.11E-01	1.83E-01, 5.27E-01	1.11E-01	8.31E-03, 1.50E-01	3.70E-02, 2.86E-01
9028	18	13	5.60E-02	1.15E-01, 4.48E-01	1.20E-02, 2.15E-01	0.00E+00	2.23E-02, 2.44E-01	0.00E+00, 1.31E-01
9020	20	0	0.00E+00	5.13E-05, 2.73E-02	0.00E+00, 1.19E-01	0.00E+00	9.74E-07, 7.19E-03	0.00E+00, 1.19E-01
6252	20	3	5.00E-02	4.56E-03, 1.07E-01	1.10E-02, 1.96E-01	0.00E+00	3.67E-04, 3.90E-02	0.00E+00, 1.19E-01
6271	20	4	5.00E-02	8.35E-03, 1.34E-01	1.10E-02, 1.96E-01	0.00E+00	8.16E-04, 5.30E-02	0.00E+00, 1.19E-01
6236	21	1	0.00E+00	4.36E-04, 5.03E-02	0.00E+00, 1.14E-01	0.00E+00	1.80E-05, 1.53E-02	0.00E+00, 1.14E-01
6266	21	4	0.00E+00	7.99E-03, 1.28E-01	0.00E+00, 1.14E-01	0.00E+00	7.29E-04, 4.97E-02	0.00E+00, 1.14E-01
9041	21	7	0.00E+00	2.60E-02, 2.11E-01	0.00E+00, 1.14E-01	0.00E+00	3.22E-03, 9.16E-02	0.00E+00, 1.14E-01
9102	23	8	2.17E-01	2.98E-02, 2.16E-01	1.10E-01, 3.84E-01	1.30E-01	3.69E-03, 9.58E-02	5.30E-02, 2.85E-01
9036	24	6	4.20E-02	1.58E-02, 1.59E-01	9.00E-03, 1.67E-01	0.00E+00	1.72E-03, 6.51E-02	0.00E+00, 1.01E-01
9009	25	1	0.00E+00	3.74E-04, 4.23E-02	0.00E+00, 9.80E-02	0.00E+00	1.46E-05, 1.27E-02	0.00E+00, 9.80E-02
9027	25	6	8.00E-02	1.51E-02, 1.53E-01	2.70E-02, 2.15E-01	0.00E+00	1.55E-03, 6.15E-02	0.00E+00, 9.80E-02
6221	26	9	1.54E-01	3.13E-02, 2.12E-01	7.10E-02, 3.02E-01	7.70E-02	4.07E-03, 9.41E-02	2.60E-02, 2.08E-01

Continued on next page

Table D–continued from previous page

Village	n	Z	$Prop_{8k}$	95% PI_{8k}	95% CI_{8k}	$Prop_{30k}$	95% PI_{30k}	95% CI_{30k}
9086	28	3	7.10E-02	3.02E-03, 7.57E-02	2.40E-02, 1.94E-01	3.60E-02	2.11E-04, 2.63E-02	8.00E-03, 1.45E-01
6248	28	6	7.10E-02	1.29E-02, 1.35E-01	2.40E-02, 1.94E-01	0.00E+00	1.29E-03, 5.36E-02	0.00E+00, 8.80E-02
9006	28	7	3.60E-02	1.74E-02, 1.55E-01	8.00E-03, 1.45E-01	0.00E+00	1.87E-03, 6.38E-02	0.00E+00, 8.80E-02
9050	28	7	3.60E-02	1.73E-02, 1.56E-01	8.00E-03, 1.45E-01	0.00E+00	1.91E-03, 6.39E-02	0.00E+00, 8.80E-02
9043	29	7	3.40E-02	1.63E-02, 1.50E-01	8.00E-03, 1.41E-01	0.00E+00	1.77E-03, 6.11E-02	0.00E+00, 8.50E-02
9071	29	7	3.40E-02	1.67E-02, 1.49E-01	8.00E-03, 1.41E-01	0.00E+00	1.77E-03, 6.12E-02	0.00E+00, 8.50E-02
9040	30	6	3.30E-02	1.16E-02, 1.25E-01	7.00E-03, 1.36E-01	0.00E+00	1.13E-03, 4.93E-02	0.00E+00, 8.30E-02
9024	30	8	0.00E+00	2.06E-02, 1.62E-01	0.00E+00, 8.30E-02	0.00E+00	2.31E-03, 6.85E-02	0.00E+00, 8.30E-02
9063	31	0	0.00E+00	4.03E-05, 1.86E-02	0.00E+00, 8.00E-02	0.00E+00	6.83E-07, 4.49E-03	0.00E+00, 8.00E-02
6234	32	7	0.00E+00	1.45E-02, 1.35E-01	0.00E+00, 7.80E-02	0.00E+00	1.48E-03, 5.40E-02	0.00E+00, 7.80E-02
9017	32	8	3.10E-02	1.88E-02, 1.51E-01	7.00E-03, 1.29E-01	3.10E-02	2.09E-03, 6.28E-02	7.00E-03, 1.29E-01
9037	32	9	6.20E-02	2.38E-02, 1.69E-01	2.10E-02, 1.72E-01	3.10E-02	2.73E-03, 7.20E-02	7.00E-03, 1.29E-01
6220	34	7	5.90E-02	1.33E-02, 1.26E-01	2.00E-02, 1.63E-01	2.90E-02	1.34E-03, 5.03E-02	7.00E-03, 1.22E-01
9077	34	8	0.00E+00	1.71E-02, 1.42E-01	0.00E+00, 7.40E-02	0.00E+00	1.80E-03, 5.81E-02	0.00E+00, 7.40E-02
6219	34	9	5.90E-02	2.16E-02, 1.58E-01	2.00E-02, 1.63E-01	2.90E-02	2.42E-03, 6.63E-02	7.00E-03, 1.22E-01
9042	35	2	0.00E+00	9.50E-04, 4.49E-02	0.00E+00, 7.20E-02	0.00E+00	4.85E-05, 1.39E-02	0.00E+00, 7.20E-02
9076	36	3	0.00E+00	2.20E-03, 5.81E-02	0.00E+00, 7.00E-02	0.00E+00	1.36E-04, 1.90E-02	0.00E+00, 7.00E-02
9070	36	10	2.80E-02	2.41E-02, 1.65E-01	6.00E-03, 1.15E-01	0.00E+00	2.77E-03, 6.96E-02	0.00E+00, 7.00E-02
9068	37	1	0.00E+00	2.56E-04, 2.82E-02	0.00E+00, 6.80E-02	0.00E+00	8.37E-06, 7.92E-03	0.00E+00, 6.80E-02
9084	37	1	0.00E+00	2.57E-04, 2.83E-02	0.00E+00, 6.80E-02	0.00E+00	8.05E-06, 8.03E-03	0.00E+00, 6.80E-02
6270	37	7	0.00E+00	1.19E-02, 1.15E-01	0.00E+00, 6.80E-02	0.00E+00	1.13E-03, 4.47E-02	0.00E+00, 6.80E-02
9030	37	13	1.08E-01	3.75E-02, 2.07E-01	4.90E-02, 2.20E-01	5.40E-02	4.86E-03, 9.31E-02	1.80E-02, 1.51E-01
9008	38	0	0.00E+00	3.59E-05, 1.50E-02	0.00E+00, 6.60E-02	0.00E+00	5.27E-07, 3.62E-03	0.00E+00, 6.60E-02
9104	38	12	2.60E-02	3.14E-02, 1.85E-01	6.00E-03, 1.10E-01	0.00E+00	3.84E-03, 8.16E-02	0.00E+00, 6.60E-02
9057	38	14	0.00E+00	4.17E-02, 2.16E-01	0.00E+00, 6.60E-02	0.00E+00	5.46E-03, 9.83E-02	0.00E+00, 6.60E-02
9069	39	1	0.00E+00	2.46E-04, 2.71E-02	0.00E+00, 6.50E-02	0.00E+00	7.65E-06, 7.45E-03	0.00E+00, 6.50E-02
9045	39	11	7.70E-02	2.57E-02, 1.66E-01	3.10E-02, 1.78E-01	0.00E+00	3.04E-03, 7.13E-02	0.00E+00, 6.50E-02
9038	40	15	1.00E-01	4.32E-02, 2.20E-01	4.60E-02, 2.05E-01	0.00E+00	5.79E-03, 1.00E-01	0.00E+00, 6.30E-02

Continued on next page

Table D–continued from previous page

Village	n	Z	$Prop_{8k}$	95% PI_{8k}	95% CI_{8k}	$Prop_{30k}$	95% PI_{30k}	95% CI_{30k}
9011	41	1	0.00E+00	2.28E-04, 2.56E-02	0.00E+00, 6.20E-02	0.00E+00	7.28E-06, 7.06E-03	0.00E+00, 6.20E-02
6255	41	15	4.90E-02	4.14E-02, 2.13E-01	1.60E-02, 1.37E-01	0.00E+00	5.50E-03, 9.72E-02	0.00E+00, 6.20E-02
9026	41	22	2.40E-02	8.24E-02, 3.18E-01	5.00E-03, 1.02E-01	0.00E+00	1.34E-02, 1.58E-01	0.00E+00, 6.20E-02
6263	42	6	4.80E-02	7.46E-03, 8.69E-02	1.60E-02, 1.34E-01	0.00E+00	6.21E-04, 3.23E-02	0.00E+00, 6.10E-02
9014	42	9	2.40E-02	1.59E-02, 1.26E-01	5.00E-03, 1.00E-01	0.00E+00	1.62E-03, 5.10E-02	0.00E+00, 6.10E-02
9064	42	11	0.00E+00	2.31E-02, 1.53E-01	0.00E+00, 6.10E-02	0.00E+00	2.60E-03, 6.40E-02	0.00E+00, 6.10E-02
9007	42	12	0.00E+00	2.74E-02, 1.66E-01	0.00E+00, 6.10E-02	0.00E+00	3.09E-03, 7.16E-02	0.00E+00, 6.10E-02
6214	43	2	2.30E-02	7.55E-04, 3.58E-02	5.00E-03, 9.80E-02	0.00E+00	3.48E-05, 1.08E-02	0.00E+00, 5.90E-02
9049	43	5	0.00E+00	4.98E-03, 7.22E-02	0.00E+00, 5.90E-02	0.00E+00	3.78E-04, 2.59E-02	0.00E+00, 5.90E-02
9021	43	16	4.70E-02	4.40E-02, 2.15E-01	1.60E-02, 1.31E-01	0.00E+00	5.80E-03, 9.82E-02	0.00E+00, 5.90E-02
9003	44	2	0.00E+00	7.40E-04, 3.51E-02	0.00E+00, 5.80E-02	0.00E+00	3.41E-05, 1.06E-02	0.00E+00, 5.80E-02
9113	44	15	9.10E-02	3.76E-02, 1.98E-01	4.10E-02, 1.88E-01	2.30E-02	4.82E-03, 8.87E-02	5.00E-03, 9.60E-02
9099	45	15	8.90E-02	3.67E-02, 1.92E-01	4.10E-02, 1.84E-01	2.20E-02	4.62E-03, 8.62E-02	5.00E-03, 9.40E-02
9001	46	0	0.00E+00	3.22E-05, 1.27E-02	0.00E+00, 5.60E-02	0.00E+00	4.69E-07, 2.92E-03	0.00E+00, 5.60E-02
9098	46	12	2.20E-02	2.39E-02, 1.50E-01	5.00E-03, 9.20E-02	2.20E-02	2.71E-03, 6.34E-02	5.00E-03, 9.20E-02
6210	46	14	0.00E+00	3.13E-02, 1.75E-01	0.00E+00, 5.60E-02	0.00E+00	3.80E-03, 7.71E-02	0.00E+00, 5.60E-02
9016	47	0	0.00E+00	3.13E-05, 1.24E-02	0.00E+00, 5.40E-02	0.00E+00	4.50E-07, 2.86E-03	0.00E+00, 5.40E-02
9029	47	0	0.00E+00	3.18E-05, 1.24E-02	0.00E+00, 5.40E-02	0.00E+00	4.72E-07, 2.91E-03	0.00E+00, 5.40E-02
9056	48	2	2.10E-02	6.85E-04, 3.21E-02	5.00E-03, 8.80E-02	2.10E-02	2.94E-05, 9.47E-03	5.00E-03, 8.80E-02
6216	48	5	0.00E+00	4.33E-03, 6.42E-02	0.00E+00, 5.30E-02	0.00E+00	3.06E-04, 2.25E-02	0.00E+00, 5.30E-02
9058	48	20	0.00E+00	5.48E-02, 2.42E-01	0.00E+00, 5.30E-02	0.00E+00	7.76E-03, 1.13E-01	0.00E+00, 5.30E-02
9095	49	11	4.10E-02	1.84E-02, 1.28E-01	1.40E-02, 1.16E-01	2.00E-02	1.94E-03, 5.28E-02	5.00E-03, 8.60E-02
9022	50	0	0.00E+00	2.99E-05, 1.16E-02	0.00E+00, 5.10E-02	0.00E+00	4.49E-07, 2.63E-03	0.00E+00, 5.10E-02
9083	50	1	0.00E+00	1.87E-04, 2.08E-02	0.00E+00, 5.10E-02	0.00E+00	5.46E-06, 5.53E-03	0.00E+00, 5.10E-02
9033	50	8	4.00E-02	1.01E-02, 9.29E-02	1.30E-02, 1.14E-01	2.00E-02	8.90E-04, 3.59E-02	4.00E-03, 8.50E-02
9062	50	15	2.00E-02	3.13E-02, 1.71E-01	4.00E-03, 8.50E-02	0.00E+00	3.78E-03, 7.51E-02	0.00E+00, 5.10E-02
6212	51	12	5.90E-02	2.07E-02, 1.34E-01	2.40E-02, 1.38E-01	0.00E+00	2.18E-03, 5.53E-02	0.00E+00, 5.00E-02
6235	51	16	0.00E+00	3.41E-02, 1.79E-01	0.00E+00, 5.00E-02	0.00E+00	4.24E-03, 7.93E-02	0.00E+00, 5.00E-02

Continued on next page

Table D–continued from previous page

Village	n	Z	$Prop_{8k}$	95% PI_{8k}	95% CI_{8k}	$Prop_{30k}$	95% PI_{30k}	95% CI_{30k}
6247	52	5	0.00E+00	3.89E-03, 5.91E-02	0.00E+00, 4.90E-02	0.00E+00	2.65E-04, 2.02E-02	0.00E+00, 4.90E-02
9051	52	13	7.70E-02	2.29E-02, 1.42E-01	3.50E-02, 1.61E-01	7.70E-02	2.52E-03, 5.91E-02	3.50E-02, 1.61E-01
6239	53	1	0.00E+00	1.80E-04, 1.95E-02	0.00E+00, 4.90E-02	0.00E+00	4.78E-06, 5.16E-03	0.00E+00, 4.90E-02
9082	53	2	0.00E+00	5.99E-04, 2.87E-02	0.00E+00, 4.90E-02	0.00E+00	2.49E-05, 8.38E-03	0.00E+00, 4.90E-02
6260	53	7	5.70E-02	7.33E-03, 7.78E-02	2.30E-02, 1.33E-01	3.80E-02	5.80E-04, 2.86E-02	1.30E-02, 1.08E-01
9093	53	9	0.00E+00	1.17E-02, 9.76E-02	0.00E+00, 4.90E-02	0.00E+00	1.04E-03, 3.77E-02	0.00E+00, 4.90E-02
6229	53	10	7.50E-02	1.40E-02, 1.07E-01	3.40E-02, 1.58E-01	1.90E-02	1.35E-03, 4.26E-02	4.00E-03, 8.00E-02
9073	54	0	0.00E+00	2.89E-05, 1.08E-02	0.00E+00, 4.80E-02	0.00E+00	3.91E-07, 2.45E-03	0.00E+00, 4.80E-02
6208	54	6	3.70E-02	5.18E-03, 6.62E-02	1.20E-02, 1.06E-01	0.00E+00	3.87E-04, 2.34E-02	0.00E+00, 4.80E-02
9087	55	1	0.00E+00	1.69E-04, 1.87E-02	0.00E+00, 4.70E-02	0.00E+00	4.64E-06, 4.91E-03	0.00E+00, 4.70E-02
9004	55	11	3.60E-02	1.60E-02, 1.13E-01	1.20E-02, 1.04E-01	0.00E+00	1.56E-03, 4.51E-02	0.00E+00, 4.70E-02
6237	55	17	9.10E-02	3.40E-02, 1.74E-01	4.50E-02, 1.75E-01	0.00E+00	4.10E-03, 7.71E-02	0.00E+00, 4.70E-02
9107	55	19	7.30E-02	4.05E-02, 1.96E-01	3.30E-02, 1.52E-01	1.80E-02	5.25E-03, 8.82E-02	4.00E-03, 7.70E-02
6240	56	6	3.60E-02	5.01E-03, 6.30E-02	1.20E-02, 1.02E-01	0.00E+00	3.66E-04, 2.22E-02	0.00E+00, 4.60E-02
9012	56	8	0.00E+00	8.65E-03, 8.23E-02	0.00E+00, 4.60E-02	0.00E+00	7.25E-04, 3.09E-02	0.00E+00, 4.60E-02
9111	56	14	1.80E-02	2.36E-02, 1.41E-01	4.00E-03, 7.60E-02	1.80E-02	2.59E-03, 5.98E-02	4.00E-03, 7.60E-02
9066	57	1	0.00E+00	1.64E-04, 1.81E-02	0.00E+00, 4.50E-02	0.00E+00	4.45E-06, 4.73E-03	0.00E+00, 4.50E-02
6246	57	4	1.80E-02	2.24E-03, 4.40E-02	4.00E-03, 7.50E-02	1.80E-02	1.31E-04, 1.44E-02	4.00E-03, 7.50E-02
6226	57	8	3.50E-02	8.51E-03, 8.08E-02	1.20E-02, 1.01E-01	1.80E-02	7.07E-04, 3.01E-02	4.00E-03, 7.50E-02
6217	57	15	1.93E-01	2.60E-02, 1.47E-01	1.22E-01, 2.92E-01	1.05E-01	2.92E-03, 6.31E-02	5.50E-02, 1.91E-01
9089	57	17	7.00E-02	3.21E-02, 1.67E-01	3.20E-02, 1.47E-01	1.80E-02	3.90E-03, 7.36E-02	4.00E-03, 7.50E-02
6241	59	9	5.10E-02	1.01E-02, 8.66E-02	2.10E-02, 1.21E-01	0.00E+00	8.70E-04, 3.29E-02	0.00E+00, 4.40E-02
9023	59	11	1.70E-02	1.42E-02, 1.05E-01	4.00E-03, 7.20E-02	0.00E+00	1.36E-03, 4.17E-02	0.00E+00, 4.40E-02
9053	59	14	6.80E-02	2.21E-02, 1.33E-01	3.10E-02, 1.43E-01	3.40E-02	2.33E-03, 5.52E-02	1.10E-02, 9.70E-02
6267	59	17	1.70E-02	3.06E-02, 1.62E-01	4.00E-03, 7.20E-02	0.00E+00	3.56E-03, 6.99E-02	0.00E+00, 4.40E-02
9005	60	2	0.00E+00	5.28E-04, 2.49E-02	0.00E+00, 4.30E-02	0.00E+00	1.97E-05, 7.14E-03	0.00E+00, 4.30E-02
9019	60	2	0.00E+00	5.23E-04, 2.50E-02	0.00E+00, 4.30E-02	0.00E+00	1.98E-05, 7.06E-03	0.00E+00, 4.30E-02
9025	60	2	1.70E-02	5.18E-04, 2.54E-02	4.00E-03, 7.10E-02	0.00E+00	2.04E-05, 7.15E-03	0.00E+00, 4.30E-02

Continued on next page

Table D–continued from previous page

Village	n	Z	Prop_{8k}	95% PI_{8k}	95% CI_{8k}	Prop_{30k}	95% PI_{30k}	95% CI_{30k}
6243	60	9	3.30E-02	9.74E-03, 8.56E-02	1.10E-02, 9.60E-02	0.00E+00	8.62E-04, 3.20E-02	0.00E+00, 4.30E-02
9055	60	11	0.00E+00	1.41E-02, 1.03E-01	0.00E+00, 4.30E-02	0.00E+00	1.33E-03, 4.08E-02	0.00E+00, 4.30E-02
9081	60	15	6.70E-02	2.41E-02, 1.40E-01	3.00E-02, 1.40E-01	1.70E-02	2.70E-03, 5.93E-02	4.00E-03, 7.10E-02
6223	60	17	8.30E-02	2.95E-02, 1.59E-01	4.10E-02, 1.61E-01	3.30E-02	3.43E-03, 6.84E-02	1.10E-02, 9.60E-02
9060	61	2	0.00E+00	5.02E-04, 2.48E-02	0.00E+00, 4.20E-02	0.00E+00	1.95E-05, 6.93E-03	0.00E+00, 4.20E-02
9031	61	3	0.00E+00	1.16E-03, 3.30E-02	0.00E+00, 4.20E-02	0.00E+00	5.36E-05, 9.84E-03	0.00E+00, 4.20E-02
9061	61	3	0.00E+00	1.15E-03, 3.29E-02	0.00E+00, 4.20E-02	0.00E+00	5.54E-05, 9.90E-03	0.00E+00, 4.20E-02
6206	61	10	3.30E-02	1.16E-02, 9.22E-02	1.10E-02, 9.40E-02	1.60E-02	1.04E-03, 3.57E-02	4.00E-03, 7.00E-02
6249	61	21	9.80E-02	4.11E-02, 1.93E-01	5.20E-02, 1.79E-01	1.60E-02	5.28E-03, 8.81E-02	4.00E-03, 7.00E-02
6207	62	3	0.00E+00	1.13E-03, 3.21E-02	0.00E+00, 4.20E-02	0.00E+00	5.55E-05, 9.65E-03	0.00E+00, 4.20E-02
9002	62	5	0.00E+00	3.07E-03, 4.83E-02	0.00E+00, 4.20E-02	0.00E+00	1.94E-04, 1.61E-02	0.00E+00, 4.20E-02
6261	62	8	1.60E-02	7.49E-03, 7.31E-02	4.00E-03, 6.90E-02	0.00E+00	5.76E-04, 2.70E-02	0.00E+00, 4.20E-02
6251	62	27	6.50E-02	6.20E-02, 2.48E-01	2.90E-02, 1.36E-01	3.20E-02	9.04E-03, 1.18E-01	1.10E-02, 9.30E-02
6205	63	1	0.00E+00	1.51E-04, 1.64E-02	0.00E+00, 4.10E-02	0.00E+00	3.71E-06, 4.16E-03	0.00E+00, 4.10E-02
6204	64	11	6.20E-02	1.28E-02, 9.56E-02	2.80E-02, 1.32E-01	0.00E+00	1.16E-03, 3.72E-02	0.00E+00, 4.10E-02
6201	64	16	4.70E-02	2.48E-02, 1.38E-01	1.90E-02, 1.12E-01	3.10E-02	2.70E-03, 5.86E-02	1.00E-02, 9.00E-02
9114	65	10	1.50E-02	1.07E-02, 8.58E-02	3.00E-03, 6.60E-02	0.00E+00	9.48E-04, 3.29E-02	0.00E+00, 4.00E-02
6256	65	11	3.10E-02	1.26E-02, 9.38E-02	1.00E-02, 8.90E-02	3.10E-02	1.14E-03, 3.67E-02	1.00E-02, 8.90E-02
6215	66	5	1.50E-02	2.83E-03, 4.49E-02	3.00E-03, 6.50E-02	0.00E+00	1.74E-04, 1.48E-02	0.00E+00, 3.90E-02
6264	66	13	1.50E-02	1.64E-02, 1.09E-01	3.00E-03, 6.50E-02	0.00E+00	1.61E-03, 4.39E-02	0.00E+00, 3.90E-02
9088	67	16	4.50E-02	2.27E-02, 1.32E-01	1.80E-02, 1.07E-01	0.00E+00	2.46E-03, 5.50E-02	0.00E+00, 3.90E-02
9109	67	34	9.00E-02	8.16E-02, 2.91E-01	4.70E-02, 1.64E-01	1.50E-02	1.27E-02, 1.45E-01	3.00E-03, 6.40E-02
6227	68	11	4.40E-02	1.18E-02, 8.92E-02	1.80E-02, 1.05E-01	0.00E+00	1.06E-03, 3.46E-02	0.00E+00, 3.80E-02
9046	70	1	0.00E+00	1.36E-04, 1.45E-02	0.00E+00, 3.70E-02	0.00E+00	3.21E-06, 3.66E-03	0.00E+00, 3.70E-02
9079	70	1	0.00E+00	1.33E-04, 1.46E-02	0.00E+00, 3.70E-02	0.00E+00	3.21E-06, 3.68E-03	0.00E+00, 3.70E-02
9034	70	2	1.40E-02	4.38E-04, 2.13E-02	3.00E-03, 6.20E-02	1.40E-02	1.58E-05, 5.86E-03	3.00E-03, 6.20E-02
9096	71	10	1.40E-02	9.38E-03, 7.79E-02	3.00E-03, 6.10E-02	0.00E+00	7.82E-04, 2.93E-02	0.00E+00, 3.70E-02
6250	71	16	2.80E-02	2.13E-02, 1.24E-01	9.00E-03, 8.20E-02	0.00E+00	2.24E-03, 5.05E-02	0.00E+00, 3.70E-02

Continued on next page

Table D–continued from previous page

Village	n	Z	Prop_{8k}	95% PI_{8k}	95% CI_{8k}	Prop_{30k}	95% PI_{30k}	95% CI_{30k}
9015	72	7	1.40E-02	4.84E-03, 5.49E-02	3.00E-03, 6.00E-02	0.00E+00	3.30E-04, 1.90E-02	0.00E+00, 3.60E-02
9085	72	28	0.00E+00	5.28E-02, 2.17E-01	0.00E+00, 3.60E-02	0.00E+00	7.32E-03, 1.01E-01	0.00E+00, 3.60E-02
9032	73	3	2.70E-02	9.00E-04, 2.70E-02	9.00E-03, 7.90E-02	0.00E+00	3.97E-05, 7.96E-03	0.00E+00, 3.60E-02
6211	73	12	2.70E-02	1.23E-02, 9.00E-02	9.00E-03, 7.90E-02	2.70E-02	1.11E-03, 3.45E-02	9.00E-03, 7.90E-02
9010	73	12	2.70E-02	1.25E-02, 9.01E-02	9.00E-03, 7.90E-02	0.00E+00	1.12E-03, 3.50E-02	0.00E+00, 3.60E-02
9105	74	37	9.50E-02	8.04E-02, 2.85E-01	5.20E-02, 1.66E-01	1.40E-02	1.28E-02, 1.41E-01	3.00E-03, 5.80E-02
6202	75	7	0.00E+00	4.55E-03, 5.27E-02	0.00E+00, 3.50E-02	0.00E+00	3.08E-04, 1.80E-02	0.00E+00, 3.50E-02
9092	75	8	2.70E-02	5.80E-03, 5.96E-02	9.00E-03, 7.70E-02	0.00E+00	4.22E-04, 2.10E-02	0.00E+00, 3.50E-02
9097	75	19	9.30E-02	2.62E-02, 1.39E-01	5.10E-02, 1.64E-01	1.30E-02	2.91E-03, 5.90E-02	3.00E-03, 5.80E-02
6230	77	16	6.50E-02	1.89E-02, 1.13E-01	3.20E-02, 1.27E-01	1.30E-02	1.90E-03, 4.63E-02	3.00E-03, 5.60E-02
9052	77	21	5.20E-02	2.97E-02, 1.49E-01	2.40E-02, 1.11E-01	0.00E+00	3.41E-03, 6.41E-02	0.00E+00, 3.40E-02
6254	77	28	1.04E-01	4.75E-02, 2.02E-01	6.00E-02, 1.75E-01	1.30E-02	6.28E-03, 9.29E-02	3.00E-03, 5.60E-02
6262	78	17	2.60E-02	2.05E-02, 1.18E-01	9.00E-03, 7.50E-02	0.00E+00	2.11E-03, 4.85E-02	0.00E+00, 3.40E-02
9054	78	17	1.30E-02	2.07E-02, 1.18E-01	3.00E-03, 5.50E-02	0.00E+00	2.09E-03, 4.84E-02	0.00E+00, 3.40E-02
9080	79	11	1.30E-02	9.59E-03, 7.55E-02	3.00E-03, 5.50E-02	0.00E+00	7.78E-04, 2.86E-02	0.00E+00, 3.30E-02
6242	80	8	2.50E-02	5.37E-03, 5.54E-02	8.00E-03, 7.30E-02	0.00E+00	3.71E-04, 1.93E-02	0.00E+00, 3.30E-02
6222	80	9	3.80E-02	6.55E-03, 6.16E-02	1.50E-02, 9.00E-02	0.00E+00	5.03E-04, 2.21E-02	0.00E+00, 3.30E-02
6209	81	10	0.00E+00	7.81E-03, 6.74E-02	0.00E+00, 3.20E-02	0.00E+00	6.09E-04, 2.48E-02	0.00E+00, 3.20E-02
9039	81	23	2.50E-02	3.18E-02, 1.55E-01	8.00E-03, 7.20E-02	1.20E-02	3.69E-03, 6.72E-02	3.00E-03, 5.30E-02
9101	82	19	3.70E-02	2.30E-02, 1.25E-01	1.50E-02, 8.80E-02	0.00E+00	2.46E-03, 5.21E-02	0.00E+00, 3.20E-02
9074	84	14	2.40E-02	1.33E-02, 8.92E-02	8.00E-03, 6.90E-02	1.20E-02	1.19E-03, 3.47E-02	3.00E-03, 5.20E-02
6224	84	25	7.10E-02	3.48E-02, 1.62E-01	3.70E-02, 1.32E-01	2.40E-02	4.14E-03, 7.18E-02	8.00E-03, 6.90E-02
9090	85	23	9.40E-02	3.00E-02, 1.46E-01	5.40E-02, 1.59E-01	0.00E+00	3.44E-03, 6.35E-02	0.00E+00, 3.10E-02
9103	86	23	7.00E-02	2.95E-02, 1.45E-01	3.70E-02, 1.29E-01	3.50E-02	3.32E-03, 6.21E-02	1.40E-02, 8.40E-02
6218	87	11	0.00E+00	8.38E-03, 6.76E-02	0.00E+00, 3.00E-02	0.00E+00	6.58E-04, 2.50E-02	0.00E+00, 3.00E-02
9013	89	0	0.00E+00	2.06E-05, 6.56E-03	0.00E+00, 3.00E-02	0.00E+00	2.34E-07, 1.36E-03	0.00E+00, 3.00E-02
9100	90	9	1.10E-02	5.50E-03, 5.42E-02	2.00E-03, 4.80E-02	0.00E+00	3.96E-04, 1.88E-02	0.00E+00, 2.90E-02
9078	91	16	1.10E-02	1.48E-02, 9.34E-02	2.00E-03, 4.80E-02	0.00E+00	1.37E-03, 3.70E-02	0.00E+00, 2.90E-02

Continued on next page

Table D–continued from previous page

Village	n	Z	Prop_{8k}	95% PI_{8k}	95% CI_{8k}	Prop_{30k}	95% PI_{30k}	95% CI_{30k}
6225	92	4	2.20E-02	1.17E-03, 2.59E-02	7.00E-03, 6.40E-02	1.10E-02	5.45E-05, 7.75E-03	2.00E-03, 4.70E-02
6244	94	16	5.30E-02	1.43E-02, 9.01E-02	2.60E-02, 1.05E-01	1.10E-02	1.30E-03, 3.52E-02	2.00E-03, 4.60E-02
6273	95	33	9.50E-02	4.58E-02, 1.90E-01	5.60E-02, 1.56E-01	1.10E-02	5.88E-03, 8.63E-02	2.00E-03, 4.60E-02
6232	98	22	5.10E-02	2.26E-02, 1.19E-01	2.50E-02, 1.01E-01	0.00E+00	2.39E-03, 4.97E-02	0.00E+00, 2.70E-02
9072	100	17	0.00E+00	1.44E-02, 8.98E-02	0.00E+00, 2.60E-02	0.00E+00	1.31E-03, 3.51E-02	0.00E+00, 2.60E-02
9048	100	38	8.00E-02	5.31E-02, 2.08E-01	4.60E-02, 1.36E-01	1.00E-02	7.24E-03, 9.73E-02	2.00E-03, 4.40E-02
9091	103	7	0.00E+00	2.91E-03, 3.64E-02	0.00E+00, 2.60E-02	0.00E+00	1.75E-04, 1.20E-02	0.00E+00, 2.60E-02
9094	105	23	1.05E-01	2.21E-02, 1.16E-01	6.50E-02, 1.64E-01	3.80E-02	2.28E-03, 4.78E-02	1.70E-02, 8.20E-02
6238	106	28	8.50E-02	2.97E-02, 1.40E-01	5.00E-02, 1.40E-01	4.70E-02	3.35E-03, 6.09E-02	2.30E-02, 9.40E-02
9110	109	34	9.20E-02	3.90E-02, 1.67E-01	5.60E-02, 1.48E-01	2.80E-02	4.71E-03, 7.45E-02	1.10E-02, 6.70E-02
9018	111	7	1.80E-02	2.65E-03, 3.39E-02	6.00E-03, 5.30E-02	0.00E+00	1.47E-04, 1.09E-02	0.00E+00, 2.40E-02
9106	116	28	7.80E-02	2.61E-02, 1.26E-01	4.60E-02, 1.29E-01	2.60E-02	2.86E-03, 5.33E-02	1.00E-02, 6.30E-02
6245	130	17	3.80E-02	1.00E-02, 6.62E-02	1.90E-02, 7.70E-02	8.00E-03	8.09E-04, 2.49E-02	2.00E-03, 3.40E-02
9112	137	36	4.40E-02	3.07E-02, 1.37E-01	2.30E-02, 8.20E-02	7.00E-03	3.45E-03, 5.92E-02	2.00E-03, 3.20E-02
6213	150	30	2.70E-02	2.03E-02, 1.01E-01	1.20E-02, 5.80E-02	7.00E-03	2.00E-03, 4.15E-02	1.00E-03, 2.90E-02
6233	178	7	0.00E+00	1.35E-03, 1.96E-02	0.00E+00, 1.50E-02	0.00E+00	6.05E-05, 5.83E-03	0.00E+00, 1.50E-02
6231	186	30	2.70E-02	1.49E-02, 7.92E-02	1.30E-02, 5.40E-02	1.10E-02	1.33E-03, 3.14E-02	4.00E-03, 3.20E-02

Equatorial Guinea

Table E: Model-based 95% prediction intervals (PI) for T, the proportion of individuals with parasite counts greater than 8000/ml blood the proportion of individuals with parasite counts greater than 30000/ml blood as well as the 95% confidence intervals for the true proportions based on the binomial sampling distribution of the observed numbers in each village. n, the number of individuals tested, Z, the number of individuals tested positive for *Loa Loa*, Prop_{8k} is the observed proportion with infection levels greater than 8000mf/ml blood, Prop_{30k} is the observed proportion with infection levels greater than 30000mf/ml blood

Village	n	Z	Prop_{8k}	95% PI_{8k}	95% CI_{8k}	Prop_{30k}	95% PI_{30k}	95% CI_{30k}

Continued on next page

Table E–continued from previous page

Village	n	Z	Prop_{8k}	95% PI_{8k}	95% CI_{8k}	Prop_{30k}	95% PI_{30k}	95% CI_{30k}
4855	13	2	0.00E+00	2.92E-03, 1.22E-01	0.00E+00, 1.72E-01	0.00E+00	2.26E-04, 4.51E-02	0.00E+00, 1.72E-01
4857	18	5	0.00E+00	1.61E-02, 1.82E-01	0.00E+00, 1.31E-01	0.00E+00	1.76E-03, 7.60E-02	0.00E+00, 1.31E-01
4851	19	0	0.00E+00	5.14E-05, 2.88E-02	0.00E+00, 1.25E-01	0.00E+00	9.67E-07, 7.81E-03	0.00E+00, 1.25E-01
4866	22	5	1.82E-01	1.20E-02, 1.48E-01	8.40E-02, 3.49E-01	0.00E+00	1.25E-03, 5.95E-02	0.00E+00, 1.10E-01
4861	22	6	0.00E+00	1.79E-02, 1.74E-01	0.00E+00, 1.10E-01	0.00E+00	2.02E-03, 7.25E-02	0.00E+00, 1.10E-01
4860	22	7	0.00E+00	2.41E-02, 2.01E-01	0.00E+00, 1.10E-01	0.00E+00	2.95E-03, 8.66E-02	0.00E+00, 1.10E-01
4843	35	3	2.90E-02	2.27E-03, 5.94E-02	6.00E-03, 1.18E-01	0.00E+00	1.45E-04, 2.00E-02	0.00E+00, 7.20E-02
4853	36	7	0.00E+00	1.22E-02, 1.18E-01	0.00E+00, 7.00E-02	0.00E+00	1.22E-03, 4.69E-02	0.00E+00, 7.00E-02
4858	37	2	0.00E+00	9.27E-04, 4.20E-02	0.00E+00, 6.80E-02	0.00E+00	4.42E-05, 1.30E-02	0.00E+00, 6.80E-02
4848	37	6	0.00E+00	8.72E-03, 9.93E-02	0.00E+00, 6.80E-02	0.00E+00	7.84E-04, 3.79E-02	0.00E+00, 6.80E-02
4847	38	3	0.00E+00	2.01E-03, 5.44E-02	0.00E+00, 6.60E-02	0.00E+00	1.24E-04, 1.80E-02	0.00E+00, 6.60E-02
4864	44	10	0.00E+00	1.83E-02, 1.33E-01	0.00E+00, 5.80E-02	0.00E+00	1.91E-03, 5.43E-02	0.00E+00, 5.80E-02
4844	44	13	6.80E-02	2.91E-02, 1.70E-01	2.80E-02, 1.59E-01	0.00E+00	3.46E-03, 7.41E-02	0.00E+00, 5.80E-02
4852	46	11	0.00E+00	2.04E-02, 1.39E-01	0.00E+00, 5.60E-02	0.00E+00	2.14E-03, 5.72E-02	0.00E+00, 5.60E-02
4845	51	10	2.00E-02	1.50E-02, 1.12E-01	4.00E-03, 8.30E-02	0.00E+00	1.46E-03, 4.49E-02	0.00E+00, 5.00E-02
4849	56	0	0.00E+00	2.83E-05, 1.04E-02	0.00E+00, 4.60E-02	0.00E+00	3.78E-07, 2.35E-03	0.00E+00, 4.60E-02
4850	60	2	0.00E+00	5.20E-04, 2.54E-02	0.00E+00, 4.30E-02	0.00E+00	1.99E-05, 7.19E-03	0.00E+00, 4.30E-02
4859	60	3	0.00E+00	1.17E-03, 3.35E-02	0.00E+00, 4.30E-02	0.00E+00	5.70E-05, 1.02E-02	0.00E+00, 4.30E-02
4862	64	8	1.60E-02	7.23E-03, 7.07E-02	3.00E-03, 6.70E-02	0.00E+00	5.71E-04, 2.59E-02	0.00E+00, 4.10E-02
4846	83	12	0.00E+00	1.03E-02, 7.80E-02	0.00E+00, 3.20E-02	0.00E+00	8.88E-04, 2.94E-02	0.00E+00, 3.20E-02
4842	85	12	1.20E-02	1.00E-02, 7.64E-02	3.00E-03, 5.10E-02	0.00E+00	8.35E-04, 2.84E-02	0.00E+00, 3.10E-02
4863	87	9	0.00E+00	5.86E-03, 5.61E-02	0.00E+00, 3.00E-02	0.00E+00	4.18E-04, 1.97E-02	0.00E+00, 3.00E-02
4841	88	11	1.10E-02	8.30E-03, 6.73E-02	3.00E-03, 4.90E-02	0.00E+00	6.53E-04, 2.46E-02	0.00E+00, 3.00E-02
4856	94	13	3.20E-02	1.01E-02, 7.33E-02	1.30E-02, 7.70E-02	0.00E+00	8.21E-04, 2.77E-02	0.00E+00, 2.80E-02
4854	107	23	0.00E+00	2.14E-02, 1.12E-01	0.00E+00, 2.50E-02	0.00E+00	2.22E-03, 4.65E-02	0.00E+00, 2.50E-02

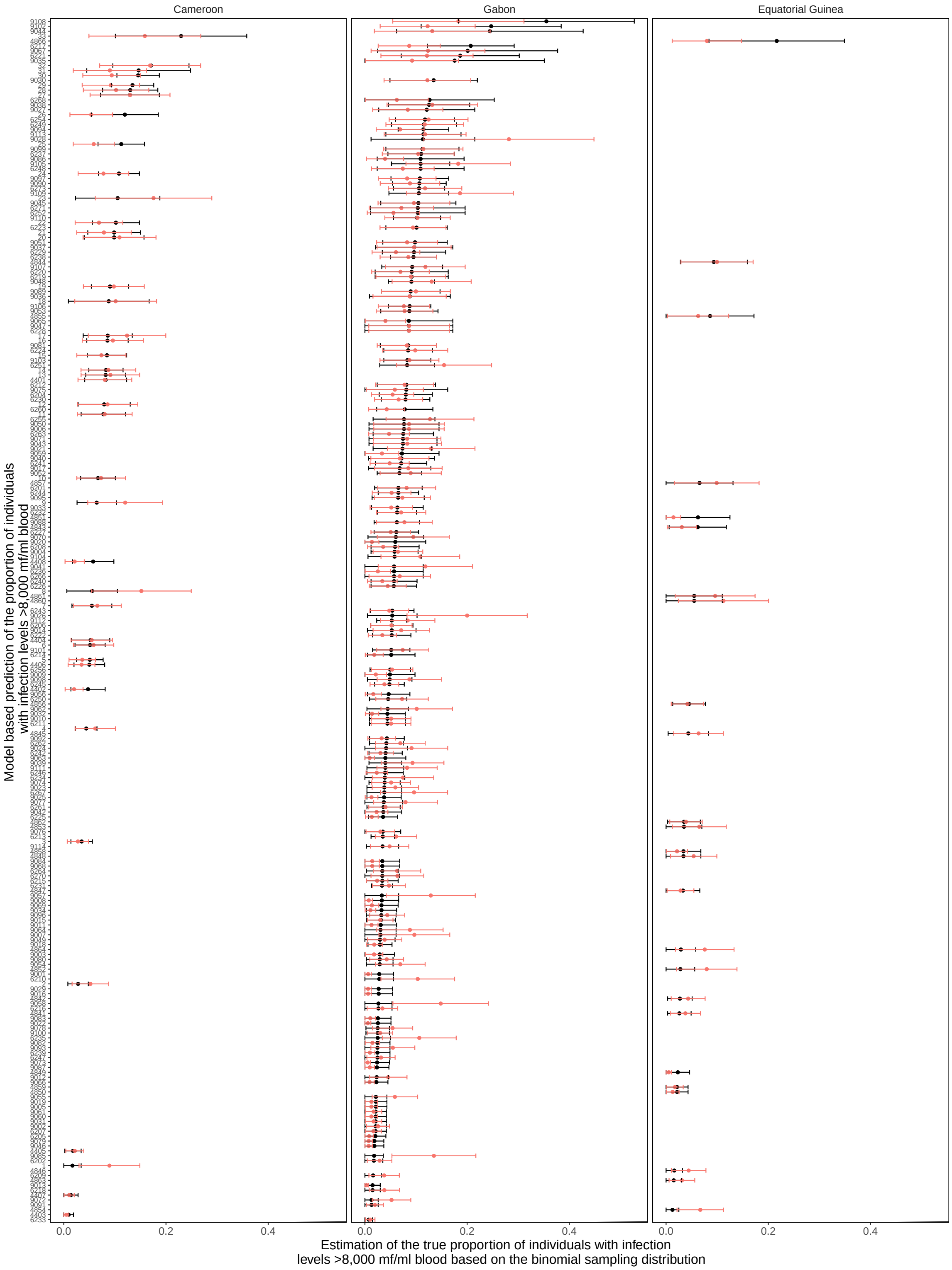

Figure E: Comparison of the proportion of individuals with more than 8,000 microfilariae per ml blood as predicted by the model based on the observed prevalence in a sample and the binomial sampling distribution based on the observed proportion in a sample. The black dots and lines are the centre and the extent of the 95% confidence interval of the binomial sampling distribution. The red dots and lines are the centre and extend of the 95% predicted interval.

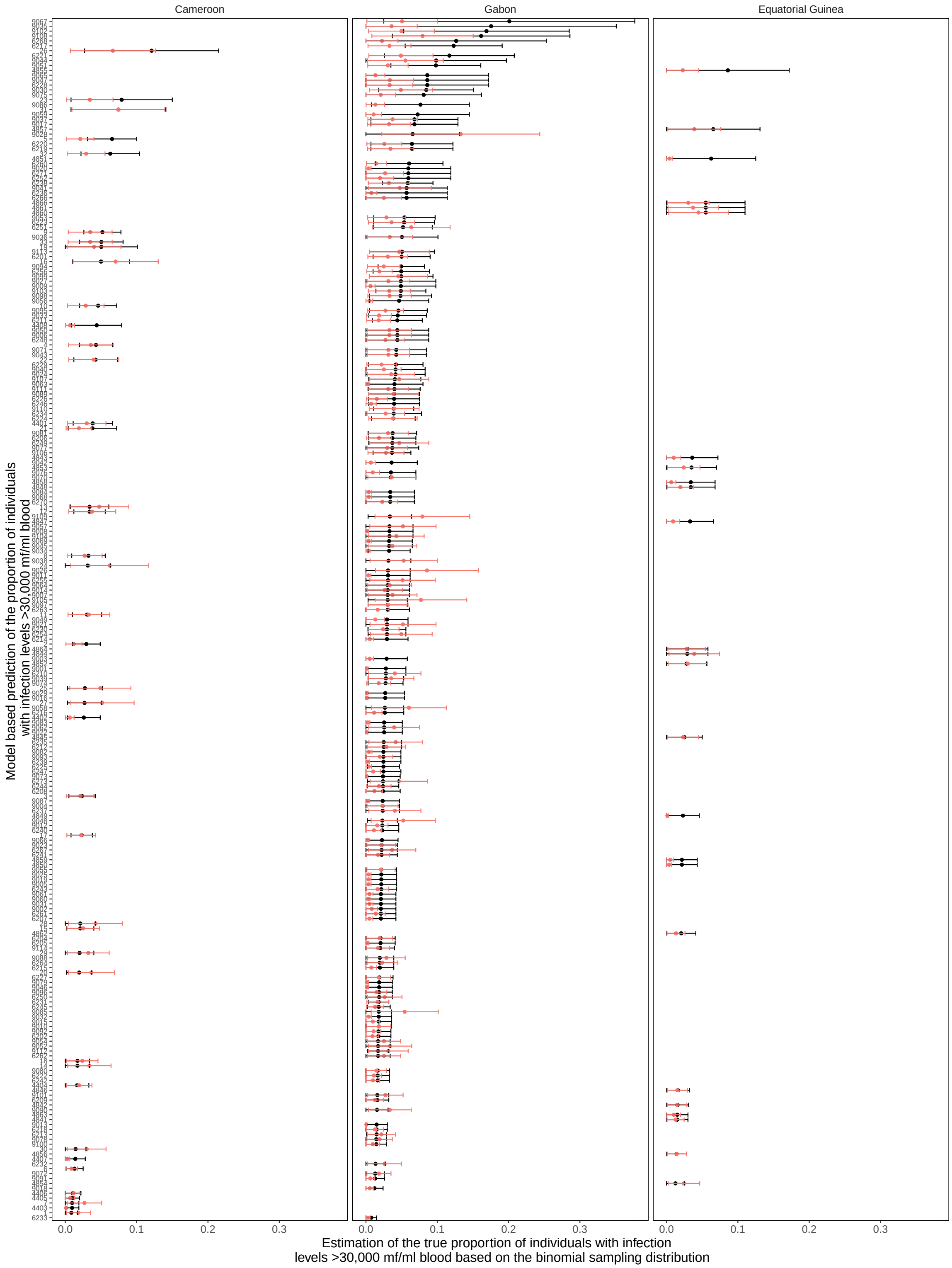

Figure F: Comparison of the proportion of individuals with more than 30,000 microfilariae per ml blood as predicted by the model based on the observed prevalence in a sample and the binomial sampling distribution based on the observed proportion in a sample. The black dots and lines are the centre and the extent of the 95% confidence interval of the binomial sampling distribution. The red dots and lines are the centre and extend of the 95% predicted interval.
